# Tropical psychotria plants are a rich source for peptide inhibitors of human prolyl oligopeptidase

**DOI:** 10.1007/s13659-026-00598-z

**Published:** 2026-04-14

**Authors:** Roland Hellinger, Paula Schwarz, Jonathan Dieringer, Carina Ebermann, Kirtikumar B. Jadhav, Markus Muttenthaler, Christian W. Gruber

**Affiliations:** 1 Center for Physiology and Pharmacology, Medical University of Vienna, 1090 Vienna, Austria; 2 Institute of Biological Chemistry, Faculty of Chemistry, University of Vienna, 1090 Vienna, Austria; 3 Institute for Molecular Bioscience, The University of Queensland, Brisbane, 4072 Australia

**Keywords:** Prolyl oligopeptidase, Plant peptide discovery, Peptide-protease inhibition, Cystine-knot peptide, Cyclotide, Peptidomics

## Abstract

Peptides from natural sources have often served as valuable leads in drug discovery. Plant-derived protease inhibitors are a notable class, yet their distribution, diversity, and targets remain underexplored. Here, eleven tropical Psychotria species were screened for cyclic cysteine-rich peptides, with extracts showing concentration-dependent inhibition of human prolyl oligopeptidase (POP). Peptidomics combining mass spectrometry and transcriptome mining revealed multiple inhibitory peptides. From *Psychotria solitudinum*, which contained 37 peptides, a novel peptide (psysol 3) was purified and sequenced. Its synthetic analogue inhibited POP with an IC_50_ of ~ 1.3 µM. Sequence analysis and synthetic probes identified loop 3 as the inhibitory motif. Psysol 3 is a new probe for POP pharmacology and future structure–activity studies.

**Supplementary Information:**

The online version contains supplementary material available at 10.1007/s13659-026-00598-z.

## Introduction

Plants are an extraordinary source for natural products, with plant-derived peptides emerging as valuable bioactive molecules, pharmacological probes, and drug candidates. There are many cysteine-stabilized peptides, including defensins, snakins, thionins, hevein-like, knottins, lipid transfer, and cyclotides [1]. Although their diversity and distribution remain underexplored, advances in peptidomics workflows now enable high-throughput discovery of novel peptides from plants [2–4]. At least twelve structurally distinct families of peptide-based protease inhibitors (peptide-PI) have been described [5]. These block clinically relevant proteases and have served as templates for drug development [5], as exemplified by angiotensin-I converting enzyme (ACE) inhibitors derived from snake venom peptides [6, 7] or thrombin inhibitors based on hirudin [8]. Protease-inhibiting peptides are particularly attractive as drug candidates because their secondary structure and cysteine connectivity confer strong resistance to proteolysis [9]. Beyond classical competitive inhibition (Laskowski mechanism), natural peptide inhibitors also act via alternative modes [5, 10], such as leech-derived peptides and their analogs (*e.g.* bivalirudin), which bind to thrombin exosites independently of antithrombin-III [11].

The human proteasome comprises serine proteases (~ 200), metalloproteinases (~ 200), cysteine proteases (~ 169), threonine proteases (~ 30) and aspartic acid proteases (~ 25) [5, 9]. These enzymes regulate key physiological systems and contribute to disease pathogenesis. Accordingly, proteases are important drug targets across multiple indications, including hypertension (ACE, renin), cardiovascular disease (neprilysin, etc.), antiviral (HIV protease), anticoagulation (thrombin), diabetes (dipeptidyl peptidase-4), or cancer (matrix metalloproteases) [9, 12]. Proteases are increasingly recognized as therapeutic targets [13], yet many remain underexplored [9]. Their moderate druggability and propensity for resistance mutations pose translational challenges, similar to those encountered with kinase inhibitors [13]. The COVID-19 pandemic highlighted the therapeutic value of viral protease inhibition [14]. Future progress is expected from artificial intelligence (AI), deep-learning models, and rational design strategies, particularly for macrocyclic and cysteine-rich peptides [15].

This study focused on human prolyl oligopeptidase (POP or PREP, EC 3.4.21.26), an 81 kDa serine protease of the S9A family (prolyl endopeptidases), often considered a drug target for different diseases, such as inflammatory disorders, neurodegeneration or parasite infections [16]. POP is structurally distinct from chymotrypsin-like proteases, with rare proline-specific endopeptidase activity [17]. The porcine POP structure revealed its two-domain organization [18], while for the human isoform only one closed-state X-ray structure exists, and complementary studies have yielded limited insights [19]. POP is expressed in neurons, liver, kidney, testis, thymus, and peripheral blood cells [18, 20–22], yet its precise physiological roles remain unclear. Initially identified as an oxytocin (OT)-cleaving enzyme [23, 24], POP is now recognized for degrading neuropeptides and peptide hormones (< 30 amino acids). Elevated POP activity has been linked to neurodegeneration, particularly Parkinson’s disease, prompting the development of specific inhibitors. The small molecule peptidomimetic KYP-2047 improved pathology in Parkinson’s mouse models [25]. However, clinical validation of POP inhibitors is still lacking, largely due to limited structure–activity relationship and pharmacological data.

Our laboratory recently identified psysol 2, a selective POP inhibitor from *Psychotria solitudinum Standl.*, which did not affect other serine proteases, such as trypsin and chymotrypsin [26]. The genus Psychotria belongs to the Rubiaceae family (subfamily Rubioideae, tribe Psychotrieae) comprises ~ 1600 species [27], many of which remain unexplored for peptide expression. In this study, we investigated the peptidomes of eleven tropical Psychotria species to identify novel cyclotides and evaluated their inhibition activity of human POP. Peptide-enriched extracts were screened for inhibitory activity, and their peptide content was characterized by mass spectrometry (MS) and transcriptome analysis. Protease inhibition activity correlated with the peptide content within the extracts. Bioassay-guided isolation of *P. solitudinum* enabled the isolation of a novel inhibitor, psysol 3, which was sequenced by *de novo* annotation of MS/MS spectra and tested for POP inhibition potency.

## Materials and methods

### Materials

Ammonium hydrogen carbonate (biochemical grade), acetonitrile (ACN) (HPLC gradient grade), Kyp-2047 ((2*S*)-1-[[(2*S*)-1-(1-oxo-4-phenylbutyl)-2-pyrrolidinyl]carbonyl]-2-pyrrolidinecarbonitrile), formic acid (FA) (LC–MS grade), bovine trypsin (sequencing grade), dithiothreitol (DTT) (biochemistry grade), α-cyano-hydroxy cinnamic acid, trypsin (sequencing grade), triisopropyl silane (TIPS) and iodoacetamide (IAA) were from Sigma Aldrich (St. Louis, United States). Chymotrypsin and endoprotease GluC (sequencing grade) were from New England Biolabs GmbH (Frankfurt am Main, Germany). Dichloromethane (DCM), (HPLC and LC–MS grade), N,N-diisopropylethylamine (DIPEA), methanol (MeOH) (HPLC synthesis or gradient grade), water (LC–MS grade), diethylether and 2-propanol (LC–MS grade) were from ChemLab (Bensheim, Germany). Trifluoroacetic acid (TFA), tris-(hydroxymethyl)-aminomethane (Tris), sodium chloride (NaCl), acetic acid, reduced or oxidized glutathione (GSH or GSSG, respectively) were from Carl Roth (Karlsruhe, Germany). Human oxytocin (OT) and Z-Gly-Pro-AMC were from Bachem (Bubendorf, Switzerland).

### Plant peptide extraction

Psychotria species were collected in different regions near the tropical research station La Gamba in Costa Rica. The deposition IDs and verification of the studied plant species have been reported elsewhere [28]. In detail: *Psychotria pilosa* Ruiz & Pav., Harald Greger, HG1907087 (WU); *Psychotria mortoniana* Standl., HG2407087 (WU); *Psychotria solitudinum* Standl., HG2607083 (WU); *Psychotria poeppigiana* Müll. Arg., HG2607081 (WU); *Psychotria erecta* (Aubl.) Standl. & Steyerm. = Syn: *Ronabea latifolia* Aubl., HG19070810-2011 (WU); *Psychotria polyphlebia* Donn.Sm. = Syn: *Notopleura polyphlebia* (Donn. Sm.) C. M. Taylor, HG2907084 (WU); *Psychotria elata* (Sw.) Hammel, HG2407081 (WU); *Psychotria capitata* Ruiz & Pav., HG24070811 (WU); *Carapichea ipecacuanha* (Brot.) L. Andersson, W1922-0008476 (NHM); *Psychotria borucana* (A.Molina) C.M.Taylor & W.C.Burger, HG207084 (WU). *Psychotria macrophylla* Ruiz & Pav. = Syn: *Notopleura capacifolia* (Dwyer) C. M. Taylor, WU0044587 & WU0044588. All plants were deposited at the Herbarium of the Institute of Botany, University of Vienna (WU) or the Museum of Natural History (NHM), Vienna. The specimen collection and the export of plant material were kindly permitted by the Costa Rican Ministry of Ambient and Energy under permit numbers 050-2013-SWAC, 217-2012-SWAC, and DGVS-109–2013, respectively [28]. The dried and ground plant material was extracted with DCM/MeOH 1:1 under continuous stirring for 24 h. A liquid–liquid phase separation by adding one-half aliquot of water was performed to extract the peptides into the aqueous phase. After freeze-drying, the solid was dissolved in 0.1% TFA in water and a solid-phase peptide extraction with C_18_ modified silica (Phenomenex) was performed according to established protocols [29]. The reversed-phase purified fraction corresponded to all molecules eluted within the 20 to 80% ACN in 0.1% TFA. Referred to as peptide extract, this product was freeze-dried and stored at -20 °C.

### Peptide synthesis and oxidative folding of cyclotides

The reference peptide psysol 2 was chemically prepared as previously described [26, 30]. Psysol 3 was synthesized by solid-phase peptide synthesis (SPPS) utilizing the 9-fluorenylmethoxycarbonyl (Fmoc) protecting strategy with an adopted protocol as described before [31]. 2-Chlorotrityl chloride resin (1.6 mmol/g, 200–400 mesh 1% DVB, synthesis scale of 0.1 mmol, from Iris Biotech) was used for SPPS on an automated synthesizer, Liberty Blue (CEM Cooperation, NC, United States). The first amino acid was coupled onto the resin in neat DCM, with 5 equivalents (eq.) excess of amino acid and 8 eq. excess of DIPEA for 2 h. Standard amino acid building blocks were used; the coupling reaction used the N,N'-dicyclohexylcarbodiimide (DCC)/oxyma coupling reagents with the excess ratio of 4:4:8 amino acid/coupling reagent/base to the resin scale. The full-length protected peptide was cleaved off the resin with 1% TFA (v/v) in DCM with 7 treatments, each 10 min. The collected eluate was quenched in ACN/water 1:1, the DCM was evaporated under vacuum, and the remaining solution was freeze-dried. The cyclization was achieved with 2 mM peptide in DMF with 4 equivalents (eq.) of (1-[bis(dimethylamino)methylene]-1H-1,2,3-triazolo[4,5-b]pyridinium 3-oxid hexafluorophosphate) (HATU) and 8 eq. of DIPEA for 4 h. The deprotection to the cyclic peptide was performed in TFA/TIPS/1,2-ethandithiol/water 92.5/2.5/2.5/2.5% (v/v/v/v) for 3 h. The peptide was precipitated in ice-cold diethyl ether and collected by centrifugation. The synthetic crude peptide material was treated with tris(2-carboxyethyl)phosphin-hydrochlorid (TCEP) in 0.1% TFA at 37 °C for 3 h and purified by reversed-phase high performance liquid chromatography (RP-HPLC) to isolate the reduced peptide.

For oxidative folding to obtain the native cystine-knotted peptide, cyclic reduced psysol 3 dissolved in diluted acetic acid (pH 4) was mixed at a final peptide concentration of 0.5 mg/mL with folding buffer 0.1 M ammonium hydrogen bicarbonate pH 7.8/isopropanol 1:1 (v/v) and thiol shuffling reagents, 2 mM reduced glutathione and 0.5 mM oxidized glutathione. The oxidative folding was completed after 24 h. The buffered sample was acidified to pH ~ 2 and freeze-dried. The resulting material was analyzed by MS and HPLC to ensure cysteine oxidation and formation of native psysol 3. The peptide was isolated with preparative HPLC. Co-elution experiments for the synthetic peptide materials with isolated natural psysol 2 were performed on a RP-HPLC system, and identical retention times confirmed the successful preparation.

### Synthesis of small disulfide cyclic peptides

The cyclic peptides were chemically prepared as previously described [32]. Peptide synthesis was performed via Fmoc-SPPS on a polystyrene A SH resin (100–200 mesh, loading 0.96 mmol, Rapp Polymer, Tübingen, Germany). The resin (0.05 mmol scale) was swelled in a 3:7 mixture of MeOH to DCM for 30 min and washed three times with MeOH/DCM and three times with DMF. The resin was then functionalized with the thiol-containing linker 2-(2-pyridyldithio)ethylamine (4 eq., Sigma-Aldrich), which was dissolved in MeOH/DCM, and added to the resin together with DIPEA (4 eq.). The mixture was incubated at room temperature for 3 h, after which the resin was washed three times with MeOH/DCM and three times with DMF. Fmoc-protected amino acids (4 eq.) were activated with HATU (4 eq.) and DIPEA (8 eq.) in DMF and coupled at room temperature for 1 h. Fmoc deprotection was performed after each coupling using 20% piperidine in DMF (3 × 5 min), followed by washing with DMF. After completion of the sequence, Trt-protected 3-mercaptopropionic acid (Trt-Mpa, 4 eq.) was coupled using HATU and DIPEA under the same conditions. The resin was washed and subjected to side-chain deprotection using TFA/TIPS/H₂O (95:2.5:2.5, v/v/v) at room temperature for 1.5 h, followed by washing with DCM and DMF. For cyclative release, the dried resin was incubated overnight at room temperature with 200 µL of 150 mM DIPEA in DMSO. The supernatant was collected, and the crude peptide was precipitated with ice-cold diethyl ether, centrifuged, and washed. The pellet was dissolved in ACN/H_2_O/TFA 50/50/0.1% (v/v/v). Samples were analyzed by MALDI-TOF MS (MS) and RP-HPLC. Purification was performed by preparative RP-HPLC.

### High-performance liquid chromatography

A Dionex Ultimate 3000 HPLC system (Thermo Fisher Scientific, Germany) equipped with a pump, single wavelength detector, and autosampler was used for the study. Peptide separations were performed in the reversed-phase mode using Dichrom Kromasil C_18_ columns (250 × 20.2 mm, 10 μm; 250 × 10 mm, 5 μm; 250 × 4.2 mm, 5 μm) or a Phenomenex Kinetex C_18_ column (150 × 3 mm, 2.1 μm) at 8, 4, 1 or 0.4 mL/min, respectively. Mobile phase (solvent) A was 0.1% (v/v) TFA in ddH_2_O and eluent (solvent) B consisted of ACN/ddH_2_O/TFA 90/10/0.09% (v/v/v). Separations were performed in linear gradients: injection hold of 5% B for 5 min, a separation gradient from 5 to 65% B (steepness 0.5, 1, 2 or 3% eluent B per min), followed by a 99% B wash and an equilibration phase to the starting condition. UV detection used the 280 nm trace specific for unsaturated chemical moieties such as tryptophan. Sample purification was either automatically, with fraction collection on an automatic fraction collector module with fixed time increments for collection (2.5 min), or manually in tubes.

### Mass spectrometry

MALDI-TOF–MS analysis of peptides was performed with an Autoflex Speed MALDI-TOF MS analyzer (Bruker Daltonics, Bremen, Germany) operating in the positive reflector mode for MS1 analysis and in LIFT mode for MS/MS experiments. A five-point internal calibration with Peptide Mix 4 from LaserBiolabs (Sophia-Antipolis, France) was used in the daily calibration routine to update the mass accuracy of the analyzer. The matrix α-cyano-hydroxy-cinnamic acid, dissolved in ACN/ddH_2_O/TFA 50/50/0.1% (v/v/v), was mixed in a ratio of 6:1 with the sample, and a 0.5 µL aliquot of the mixture was spotted on the MTP 384 ground steel target plate. Laser intensity, number of laser shots per averaged spectrum, detector gain settings, spectral and post-acquisition processing were optimized on an individual basis to acquire spectral data with high intensity and resolution. For MS/MS fragmentation experiments, selected precursors were isolated and post-source decay in a LIFT method was applied. Several fragmentation spectra were accumulated to increase global S/N for analyte signals.

### Protease inhibition assay

The full-length human prolyl oligopeptidase (NM_002726.4) was expressed in *E. coli* using the pET32a^+^ (MerckMillipore) expression system. The protein expression and purification were performed as recently described [29]. The fluorogenic peptide substrate (Z-Gly-Pro-AMC) was dissolved as a stock solution in assay buffer (20 mM Hepes, pH 7.4, 150 mM NaCl, 1 mM EDTA and 0.5 mM DTT) including 20% (v/v) isopropanol. The inhibition experiments were performed in black 96-well plates (Corning Kennebunk, USA) in a total volume of 200 µL including protease, substrate, assay buffer and peptide. Enzyme activity was measured by the obtained fluorescence signal derived from the release of the fluorophore of the Z-Gly-Pro-AMC substrate. The excitation wavelength was 360 ± 20 nm and the fluorescence emission readout was 450 ± 20 nm using an H4 Synergy plate reader (BioTek Agilent, USA). Kinetic inhibition data were recorded in three-minute intervals, and the inhibition data were calculated from the steepness of the linear initial velocity (v_t=2_-v_t=1_)/(t_2_-t_1_). A substrate control was used for background correction, and the maximum protease activity was defined as POP activity in assay buffer without inhibitors. The percentage of remaining protease activity was calculated as v_i_/v_0_ × 100. The data from the concentration–response experiment were transformed to a semilogarithmic concentration–response, normalized, and fitted to a four-parametric model with constraints (bottom is zero, top is 100%), to allow estimation of the half-maximal inhibitory concentration (IC_50_) by fitting the model to the experimental data.

### Blood sampling

The protocol and procedure details for blood collection, including specific collection methodology, site of blood sample collection, and amount of blood collected from each volunteer: Study participants were placed in a hygiene examination room. Whole blood was withdrawn from study participants via venous puncture using a 21G needle. A total of 10 mL was collected directly into a collection tube containing anticoagulants (i.e., sodium citrate). The blood sample was centrifuged at 600 × *g* for 10 min to pellet cellular components. Subsequently, the obtained plasma sample was snap frozen using liquid nitrogen for utilization later.

### Oxytocinase inhibition assay

Synthetic psysol 3 (13.5 µM) was preincubated with human citrated plasma (see below) or enzyme assay buffer with recombinant human POP (5 µg/mL), each spiked with 0.1 mM DTT, for 10 min. 50 µM human OT was incubated in the matrix at 37 °C for 24 h. At several time points (0, 5, 10, 20, 40, 60 min as well as 2 h, 4 h and 24 h), aliquots of 50 µL were pipetted into a reaction vessel. The proteolytic activity was quenched with 30 µL of an 8 M urea solution and with 20 µL of a 50% (w/v) TFA solution, each on ice for 10 min. The samples (two replicates) were centrifuged at 13,000 × *g* for 30 min and the clear supernatant was transferred into a micro-insert placed in an HPLC vial. The samples were injected into a Vanquish Duo HPLC system coupled with a single quadrupole ISQ-EM mass analyzer (Thermo Fisher Scientific). For separation, a C18 Kromasil 100 × 0.5 ID mm column at a flow rate of 0.125 mL/min was operated at 55 °C. Solvent C (aqueous 0.1% FA solution) was kept constant for 5 min at 5%, and increased to 60% within 20 min, followed by a wash with 99% solvent D (ACN/ddH_2_O/FA 90/10/0.1% (v/v/v)) for 5 min and an equilibration time for a total runtime of 45 min. The analytes were quantified in SIM detection mode using the mass transitions *m/z* 504.741 (M + 2H)^2+^ (OT) and *m/z* 1070.498 (M + 3H)^3+^ (psysol 3). The detection was verified by analysis of reference samples, and matrix samples were evaluated for any interfering matrix-derived mass signals. The area under the curve (AUC) was recorded for each sample, and the remaining intact peptide in percent was calculated relative to the sample at t = 0. A one-phase decay elimination kinetics was fitted to the experimental data to derive the half-life of OT in each of the matrices.

### Transcriptome and peptide gene annotation

*Psychotria solitudinum* transcriptome data set was de novo assembled using the Trinity RNA-seq assembler (14.06.2018; https://sourceforge.net/projects/trinityrnaseq/files/). The assembled transcriptome data set was evaluated for sequence similarity to the reported mature cyclotide sequence, reported at www.cybase.org.au using the tblastn search tool from the NCBI blast + service. The ‘Translate tool’ was used for a six-frame-translation of RNA to protein sequence and to search for the correct open reading frames or to assign start/stop codons. The sequences in the correct reading frame and direction are provided in the Supplementary Information Data S1. The obtained precursor amino acid sequences were manually evaluated using the ClustalOmega multiple alignment tool in comparison to the oak1 (AF393825) and cyclotide C3 (AY630565.1) precursor genes. If applicable, the ER signal domain was assigned due to conserved sequence patterns as described in [33, 34]. The obtained mature cyclotide sequences were used for the dereplication of peptides in plant extract and HPLC fractions based on the putative *m/z* values.

### Sequence analysis

The sequence logo was prepared with the online tool from https://weblogo.berkeley.edu/logo.cgi, showing a frequency plot. Sequence similarity and identity calculations between peptide sequences were performed with ‘The FastA—Sequence Similarity Searching’ as well as with ‘Emboss Stretcher’ tools available online from https://wwwdev.ebi.ac.uk/Tools.

### Structure prediction

For the structure prediction of the psysol 3 structural model, the Colab notebook https://colab.research.google.com/github/sokrypton/ColabDesign/blob/gamma/af/examples/predict.ipynb was utilized with MSA, 6 recycles, random masking, seed1, recycle 1, template (PDB: 1NB1), and all 5 models. pLDDT was used to choose from the different outputs of the highest confidence model. MarvinSketch was used for drawing, displaying and characterizing chemical structures, substructures and calculations of chemical properties (Marvin 24.3.2, Chemaxon, https://www.chemaxon.com).

## Results

### Characterization of peptide-enriched plant extracts on the enzymatic activity of human prolyl oligopeptidase

**Fig. 1:**
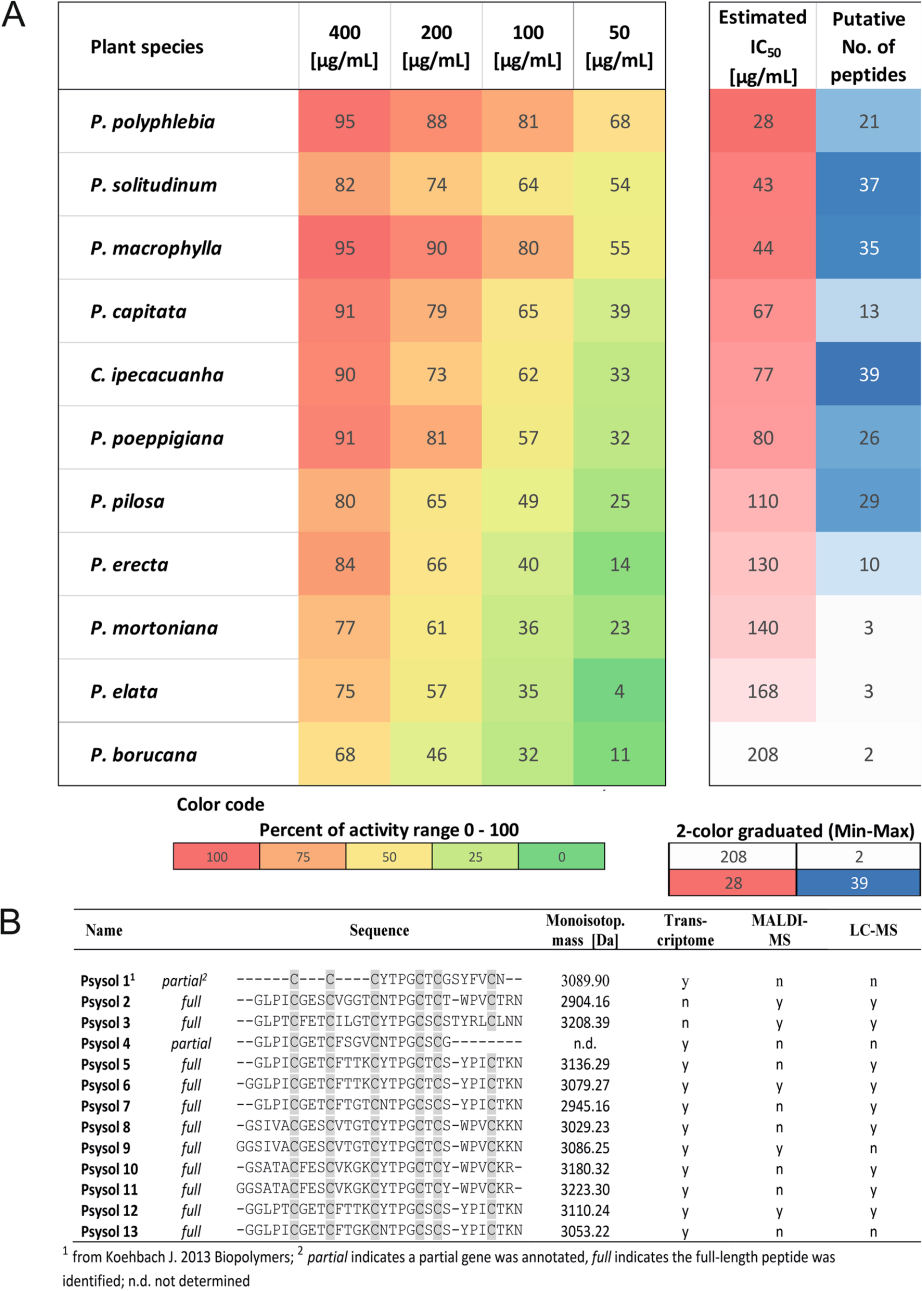
Screening of plant extracts from the Psychotria tribe toward inhibition of POP. **A** Eleven Psychotria plant extracts from *P. polypheblia, P. solitudinum, P. macrophylla, P. capitata, C. ipecacuanha, P. poeppigiana, P. pilosa, P. erecta, P. mortoniana, P. borucana,* and *P. elata*, were screened for inhibition of POP activity. From each sample, the concentrations of 50, 100, 200 and 400 µg/mL were tested to evaluate the inhibition activity of the purified crude plant extract. The inhibitory activity (%) was calculated as (1—remaining activity relative to the enzyme activity) × 100. A heat map was prepared to rank the POP inhibitory activity of the plant samples in comparison with the number of identified peptide *m/z* in the samples. The calculated IC_50_ is derived from a non-linear curve fit, as shown in Figure S1B. Data are expressed as the mean ± standard deviation (SD) of three independent experiments. Color code: Percent of relative activity range (left); 2-color graduated from min. to max. value of the data range (right). **B** Further information on the identified peptides from the transcriptome analysis of *P. solitudinum* is provided. From the annotated cyclotide precursor genes, the mature peptide sequences were extracted by identifying putative processing sites. The sequences were used to calculate putative molecular weights, and the data were matched with mass spectrometry analysis to confirm the assignment (n: no, not detected; y: yes, detected)

The Psychotria tribe (Rubiaceae) comprises over 2000 species [28, 35, 36], many of which remain phytochemically unexplored. Here, eleven representative species were selected for screening, i.e. *P. capitata, P. borucana, P. elata, P. ipecacuanha* (reclassified as *C. ipecacuanha*), *P. macrophylla, P. mortoniana, P. erecta*, *P. pilosa, P. poeppigiana, P. polyphlebia* and *P. solitudinum*. Previous reports identified cysteine-rich peptides or cyclotides in *P. solitudinum, P. capitata, P. poeppigiana, P. pilosa, C. ipecacuanha and P. elata* [26, 37], whereas *P. erecta*, *P. macrophylla* and *P. borucana* have not been investigated. Cyclotides from *P. solitudinum*, *P. capitata* and *P. poeppigiana* were earlier shown to inhibit POP [26], making them useful reference species. Samples were harvested near the tropical rainforest research station ‘La Gamba’, Costa Rica [28]. Peptide-enriched extracts were prepared using established protocols [29]: dried plant material was ground and extracted with DCM/MeOH (1:1), followed by H_2_O addition to separate peptides into the methanolic aqueous phase. Solid-phase extraction on C_18_-modified silica, further enriched mid-polar peptide fractions, yielding the so-called “peptide-enriched” extracts. The peptide-enriched extracts were tested for POP inhibition at 50–400 µg/mL (Fig. 1A). All 11 species inhibited POP activity in a concentration-dependent manner. IC_50_ values determined by nonlinear regression exhibited the strongest activity for *P. polyphlebia* (27.6 ± 1.6 µg/mL), followed by *P. solitudinum* (42.7 ± 2.5 µg/mL) and *P. macrophylla* (44.3 ± 1.3 µg/mL). Moderate activity was observed for *P. capitata* (66.5 ± 1.2 µg/mL), *C. ipecacuanha* (77.4 ± 1.3 µg/mL), *P. poeppigiana* (79.6 ± 4.9 µg/mL), while weaker activity was recorded for *P. pilosa* (110.2 ± 1.3 µg/mL), *P. erecta* (129.7 ± 1.1 µg/mL), *P. mortoniana* (140.2 ± 1.4 µg/mL), *P. elata* (167.5 ± 2.2 µg/mL) and *P. borucana* (208.4 ± 1.4 µg/mL) (Fig. 1A and Figure S1A, B). These values were slightly higher than those previously reported for *Allexis spp.* extracts [29], but comparable to earlier data for *P. capitata, P. poeppigiana* and *P. solitudinum* [26].

### Peptidomics analysis of Psychotria extracts

Several Psychotria species of this study, including *P.* *capitata*, *P. poeppigiana*, *P. mortoniana*, *P. elata*, *C. ipecacuanha*, *P. brachiata*, *P. suerensis*, *P. deflexa*, *P. chiriquiensis*, *P. goldmanii* and *P. punctata* are reported to produce cyclic cystine-knot (CCK) peptides [26, 28]. Consistent with earlier studies, we confirmed the presence of previously described peptides in extracts of *P. solitudinum*, *P. poeppigiana, P. capitata, P. elata* and *C. ipecacuanha*. Mass signals in the range of *m/z* 2000–4000 indicated cysteine-rich peptides or cyclotides [28, 36]. In contrast, only few signals were observed in *P. borucana, P. erecta, and P. mortoniana*; despite precluding further analysis due to low peptide abundance (Figure S2). By comparison, extracts of *P. polyphlebia, P. macrophylla* and *P. pilosa* had at least five detectable signals between *m/z* 2100 to 3500. High-sensitivity nanoflow LC–MS allowed the detection of 35 peptides in *P. macrophylla*, 37 in *P. solitudinum* and 39 in *C. ipecacuanha*. Across the eleven Psychotria species, POP inhibitory activity correlated with peptide content (Fig. 1A). Given its strong activity and abundant peptide signals (Figure S2A), *P. solitudinum* was selected for detailed analysis. Transcriptome analysis and MALDI-MS fingerprinting [28, 34] uncovered three full and three partial cyclotide precursor genes (Suppl. Data S1). Dereplication by MS confirmed seven putative sequences matching MS1 *m/z* values (Fig. 1B, Table S1). In total, 40 peptides were identified in *P. solitudinum*, including 12 with previously unreported amino acid sequences [28] (Table S2). These findings highlight Psychotria particularly, the Palicourea tribe within *Rubiaceae*, as an important source for cyclic cysteine-rich peptides [27, 28].

### Bioassay-guided isolation of psysol 3 from *P. solitudinum*

**Fig. 2:**
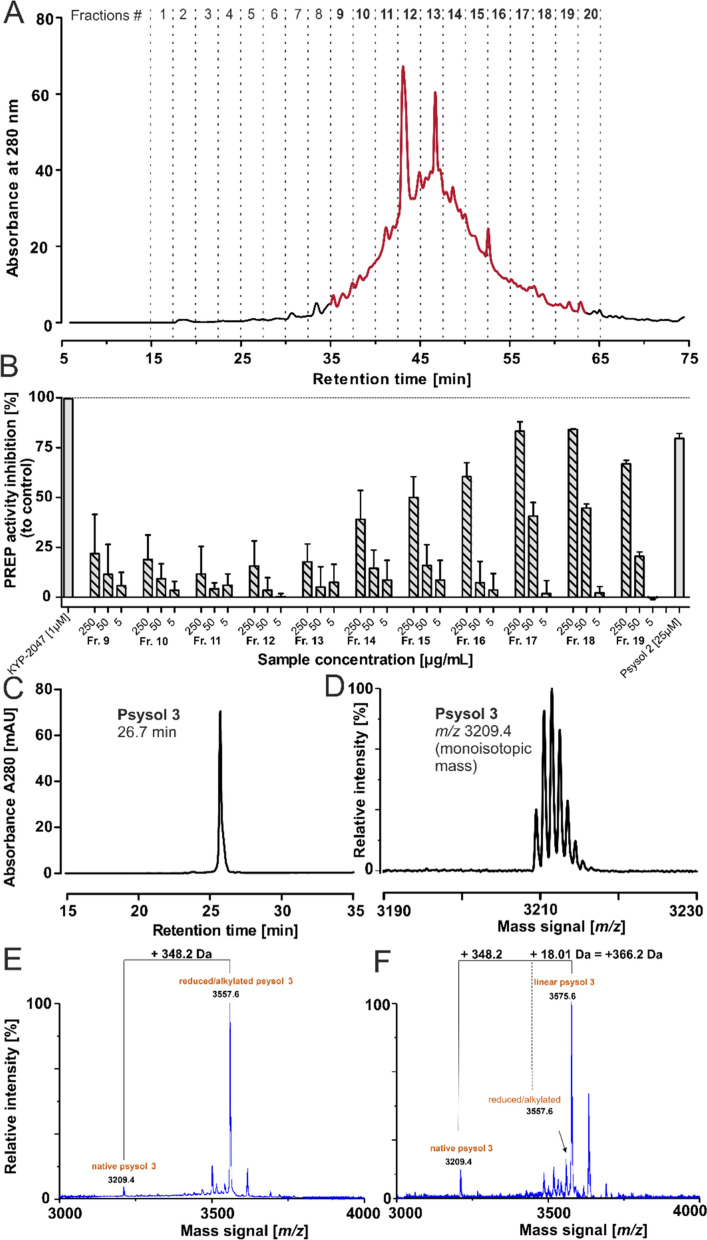
Activity-guided isolation of novel peptide POP inhibitors from *P. solitudinum*. **A** The C_18_ purified extract sample of *P. solitudinum* was fractionated by preparative HPLC using a gradient of 1% solvent B per min. Twenty fractions were collected for evaluation of the POP inhibition activity. The freeze-dried fraction materials were tested for peptide mass signals and fractions 9 to 20 showed signals in the expected range of 2500–4000 Da. The peptide-containing fractions 9–19 were assayed for inhibition of proline oligopeptidase activity in three concentrations: 5, 50, 250 µg/mL, each for three independent experiments. The inhibitory activity (%) was calculated as (1—remaining activity relative to the enzyme activity) × 100. The specific protease inhibitor KYP-2047 was tested at 1 µM, and the synthetic peptide psysol 2 was used at 25 µM. The data are reported as the mean ± SD of three independent experiments, each performed in duplicate. **C** From the most active fractions (18 & 19), a peptide was isolated with ≥ 95% purity as indicated by HPLC–UV analysis at 280 nm absorbance. **D** The *m/z* 3209.4 was detected and the peptide was denoted as psysol 3. **E** The peptide was tested for the presence of a cyclic cystine knot motif. First, a reduction and alkylation with iodoacetamide was performed, and the *m/z* trace of the converted material indicated a mass shift of + 348.2 Da. **F** The obtained precursor was incubated with endoproteinase GluC for 18 h and a further mass shift of + 366.2 Da compared to the native mass signal was recorded, which provided evidence for a cyclic cystine-rich peptide

**Fig. 3:**
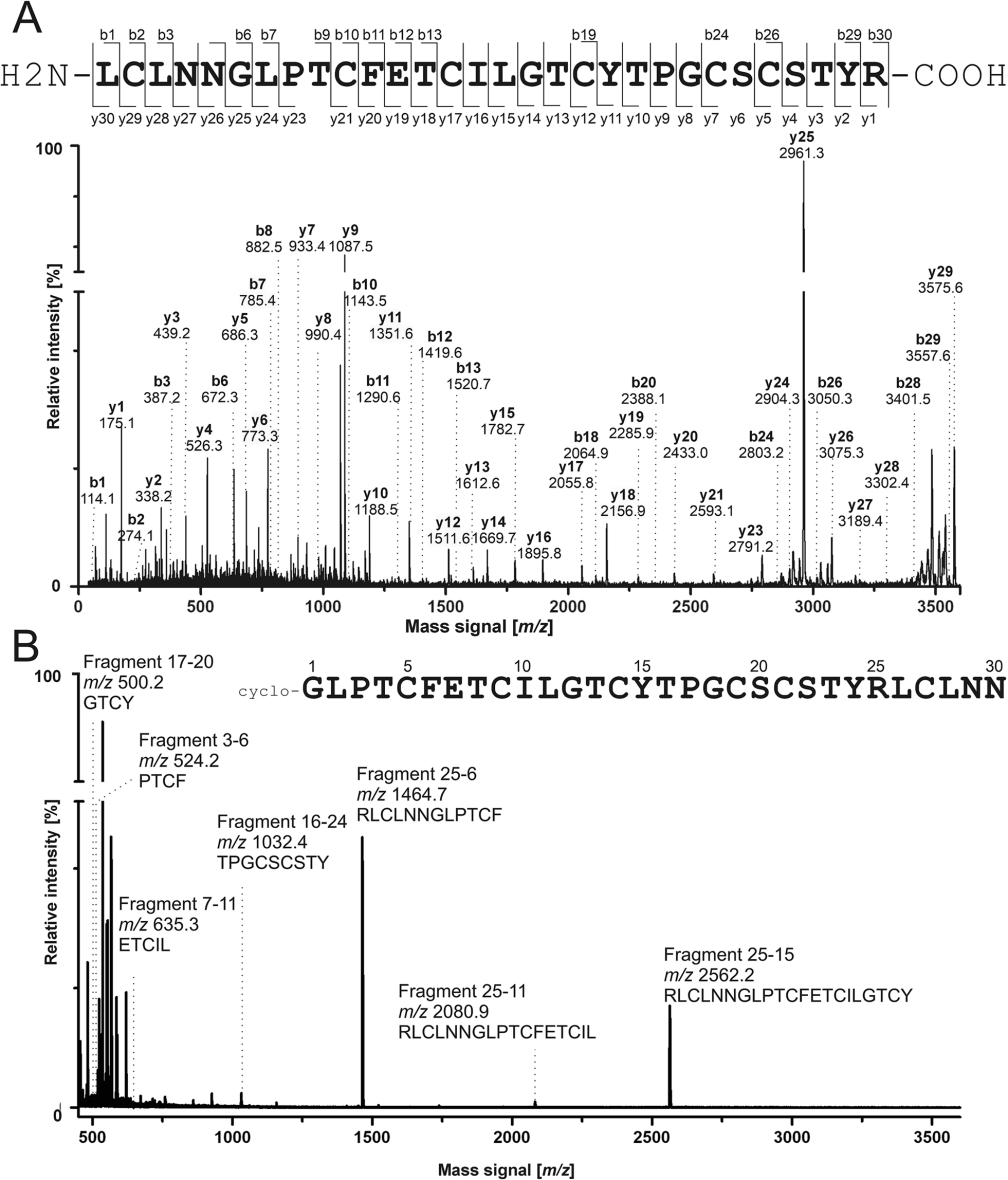
De novo peptide sequence annotation of psysol 3. **A** The purified psysol 3 was treated with dithiothreitol and sulfhydryl groups were derivatized with iodoacetamide. The resulting peptide was incubated with trypsin for a proteolysis experiment. MALDI-MS analysis showed a single proteolytic fragment with *m/z* 3575.5. This precursor was selected for an MS/MS fragmentation experiment using a MALDI-MS/MS-TOF system. The obtained peptide fragmentation spectrum was applied for de novo peptide sequence annotation to elucidate a putative sequence for psysol 3. The determined amino acid sequence for psysol 3 is shown on the top (N- to C-terminal). The identified y- and b-ions of the main fragmentation series are indicated at the sequence and in the spectrum. The y-ion series covered 29/30 signals, whereas b-ions were found for 15/30 residues. **B** Similarly, the *S*-acetamidomethylated peptide precursor was incubated with chymotrypsin. The spectrum shows the data obtained in an incubation at 37 °C for three hours. The observed mass signals were matched to theoretical chymotryptic fragments, which were calculated based on the identified sequence

The peptide-enriched extract of *P. solitudinum* was subjected to bioassay-guided fractionation to isolate a novel POP inhibitor. Iterative preparative HPLC separation produced twenty fractions, which were analyzed both chemically and in POP inhibition assays **(**Fig. 2A**)**. Fractions 1–10 and 20, contained negligible peptide amounts (< 1 mg) and were excluded from bioassays (Figure S2, Table S2). Fractions 9–19 were tested at 5, 50 and 250 µg/mL **(**Fig. 2B**)**. Fractions 9–13 exhibited modest activity (< 25% at the 250 µg/mL), whereas fractions 14–19 displayed progressively stronger inhibition, with fractions 17–19 achieving ~ 90% inhibition, comparable to 25 µM synthetic psysol 2 [26]. POP inhibitory activity correlated with peptide abundance with fraction 16 (psysol 2), fraction 17 (multiple peptides), and fractions 18–19 (single major peptide with *m/z* 3209.4) being the most active (Figure S3). The major unidentified peptide in fractions 18–19 was isolated to purity (~ 1 mg, 95% purity by HPLC–UV at 280 nm) and designated psysol 3 **(**Fig. 2C-D**)**. The peptide [M + nH]^n+^ mass signals used for the analysis are summarized in Table 1. MS analysis confirmed the presence of the CCK motif [29]. Chemical reduction and S-alkylation shifted the mass to *m/z* 3557.6 (+ 348.2 Da), consistent with six cysteines, converted to *S*-acetamides derivatives **(**Fig. 2E**)**. GluC digestion produced *m/z* 3575.6 (+ 366.2 Da), further supporting the CCK structure **(**Fig. 2F, Figure S4A). De novo sequence analysis by MS/MS of proteolytic fragments elucidated the amino acid sequence of psysol 3. Trypsin digestion of reduced and *S*-acetamidated peptide generated a precursor mass *m/z* 3575.6, yielding partial sequence information, with missing residues in loops 5 and 6 (Table 1, Fig. 3A). A GluC digestion yielded a single precursor but exhibited low fragmentation in MS/MS (Figure S4C). Sequential digestion with GluC and trypsin produced fragments with *m/z* 2156.8 and *m/z* 1459.5 (sodium adduct), of which the latter provided the missing sequence information in an MS/MS fragmentation experiment. Additionally, digestions with Gluc C and/or chymotrypsin and time-dependent cleavage patterns, enabled the assignment of isobaric residues such as Ile/Leu at positions 10–11 (Table 1; Fig. 3B). Combined MS/MS de novo sequence analysis and proteolytic mapping allowed the determination of the full-length sequence of psysol 3. Amino acid quantification via ninhydrin derivatization confirmed its composition (Table S3). The amino acid sequence of psysol 3 is: cyclo-GLPTCFETCILGTCYTPGCSCSTYRLCLNN.

**Table 1:** Summary of the peptidomics data for psysol 3

Peptide	Mass signal, (*m/z)*^a^	Corresponding sequences	Fragment position
Psysol 3	3209.2	cyclo-GLPTCFETCILGTCYTPGCSCSTYRLCLNN	1–30
Reduced variant	3215.3	n.a	1–30
*S*-acetamidated variant	3557.4	n.a	1–30
Tryptic fragment^b^	3575.4	LCLNNGLPTCFETCILGTCYTPGCSCSTYR	Full length
Endoprotease GluC fragment	3575.4	TCILGTCYTPGCSCSTYRLCLNNGLPTCFE	Full length
Digestion with GluC and trypsin in combination	2156.8 1459.8 / 1437.4^c^	TCILGTCYTPGCSCSTYR LCLNNGLPTCFE	8–25 26–7
Chymotryptic fragments^d^	2562.2 2080.9 1464.7 1032.4 635.3 524.2 500.2	RLCLNNGLPTCFETCILGTCY RLCLNNGLPTCFETCIL RLCLNNGLPTCF TPGCSCSTY ETCIL PTCF GTCY	25–15 25–11 25–6 16–24 7–11 3–6 17–20

^a^Monoisotopic [M + H]^+^ mass signals of the obtained fragments are provided in the Table

^b^The sequence of the observed fragments after proteolytic digestion and annotation is provided from the N- to the C-terminus

^c^This fragment was not observed in the mass spectrum; the corresponding Na^++^-adduct yielded low fragmentation coverage

^d^Note that not all possible chymotryptic fragments were observed in this study. n.a. not applicable

### Sequence-activity analysis of psysol 3

**Fig. 4:**
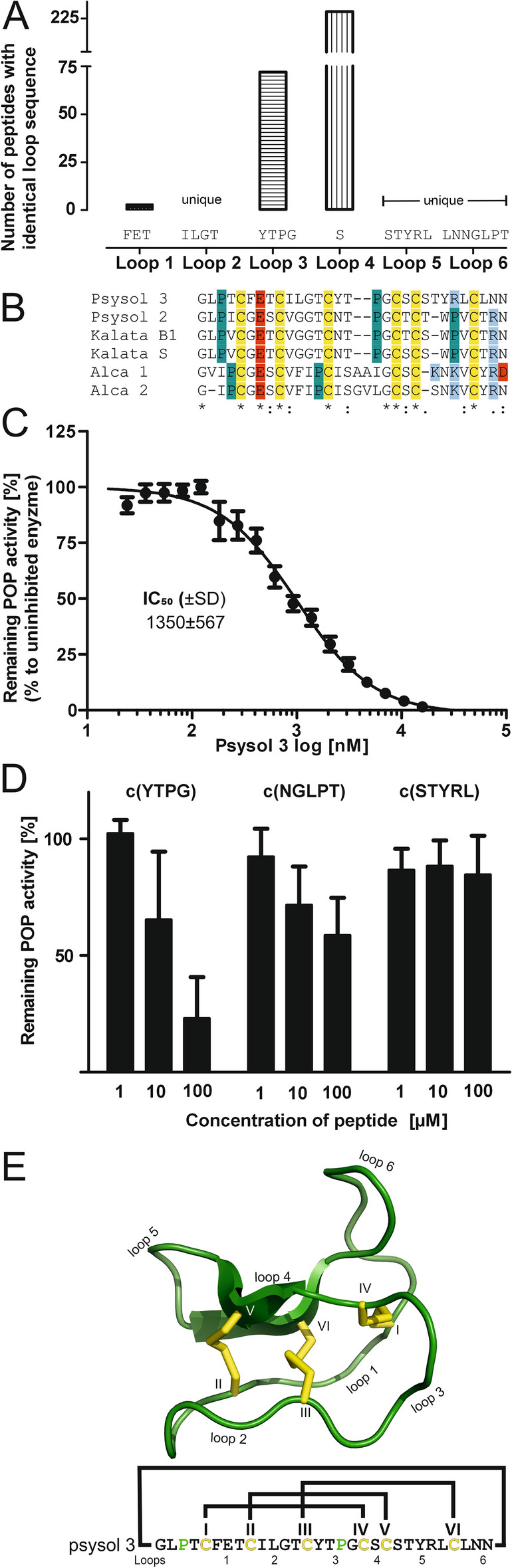
Sequence similarity analysis of psysol 3 and POP inhibition assay. **A** The primary sequence of psysol 3 was compared to all cyclotide entries published at www.cybase.au.org (> 500) in an intercysteine loop-wise manner. Psysol 3 has a unique amino acid composition in loops 2, 5 and 6, whereas loops 1, 3 and 4 are common within the cyclotide family. **B** A sequence alignment for the reported cyclotide inhibitors of human POP, which are psysol 2, -3, kalata B1, -S, and alca-1 to -2, is provided. Conserved cysteines are with a yellow background and glutamic acid as well as aspartic acid are highlighted with red, whereas basic residues, lysine and arginine, are in a blue background. Proline residues are highlighted in a petrol background. A performed sequence alignment with ClustalOmega indicated that the similarity within the inhibitor peptides is low, except for a few residues, which are important for the structure and conformation of cyclotides. **C** A concentration response experiment with psysol 3 was carried out, determining IC_50_ = 1.35 ± 0.23 µM for human prolyl oligopeptidase. The peptides’ inhibition activity data are shown as mean ± SD of five experiments. **D** The two Pro-loop– and a control sequence were prepared as cyclic probes. The three peptides were tested at 1, 10 and 100 µM, where loop 5 [c(STYRL)] did not and loop 6 [c(NGLPT)] revealed modest inhibition activity, and loop 3 [c(YTPG)] had the strongest activity in comparison. The activity derived from the intercysteine loop 3 sequences may help to localize sequence motifs representative of the inhibition activity in native cyclotide psysol 3. The experiments were performed in three independent experiments and show mean ± SD. **E** The structure of psysol 3 was predicted using AfCycDesign [15]. In the cartoon, the intercysteine loops of the cyclic peptide and cysteine residues (roman letters, I-VI) are labeled. Cysteine side chains are yellow. Below, the amino acid sequence of the peptide is provided, highlighting the disulfide connectivity with connecting lines. Prolines are green, and intercysteine loops as well as cysteine residues are labeled as in the cartoon illustration

Comparison of the experimentally derived primary sequence of psysol 3 with cyclotide entries in CyBase (www.cybase.au.org) revealed that loop 1 is present in three reported peptides, loop 2 is unique, loop 3 is common in 72 sequences, loop 4 is frequent, and loops 5 and 6 are unique (Figure S5A). A full sequence alignment of closely related cyclotides is provided in the Suppl. Data S2. The highest similarity was observed with kalata B9 (92.3%) from *Oldenlandia affinis*, while the highest identity was with mela 6 (74.1%) from *Melicytus latifolius* [37]. Sequence similarity to reported POP inhibitors was low; for example, psysol 2 shows 73.3% similarity and 56.7% identity (Fig. 4B; Suppl. Data S2). Notably, psysol 3 lacks a proline in loop 5, despite proline residues typically comprising the center of POP recognition motifs. Loops 3 and 6 are similar to other kalata-type peptides, but differ from bracelet-type peptides isolated from *Allexis spp.* (Fig. 4B; Figure S5A) [29], making psysol 3 an interesting cyclotide with three unique intercysteine loops. Psysol 3 was chemically synthesized using Fmoc-SPPS. The cyclic thiol-reduced precursor peptide was oxidatively folded to yield the native peptide (Figure S6A-C). Comparison of the synthetic peptide with native material by HPLC confirmed identical retention times, indicating correct (native) disulfide connectivity (Figure S6D, E). Synthetic psysol 3 inhibited POP with an IC_50_ of 1.35 ± 0.56 µM **(**Fig. 4C, Figure S5B), making it one of the most potent cyclotide POP inhibitors reported; for comparison, psysol 2 has an IC_50_ of ~ 25 µM [38], and alca-2 of ~ 4.4 µM [29]. To investigate loop-specific contributions to POP inhibition, small cyclic peptides derived from psysol 3 loops were synthesized: loop 3-mimetic c(YTPG), loop 5-mimetic c(STYRL, negative control), and loop 6-mimetic c(NGLPT) (Figure S7). Tested at 1, 10 and 100 µM, loop 3 c(YTPG) showed the highest activity, loop 6 c(NGLPT) had weak activity and loop 5 c(STYRL) was inactive (Fig. 4D), indicating loop 3 as the primary determinant of POP inhibition. The three-dimensional structure of psysol 3 was predicted using AlphaFold2 with cyclic peptide prediction via AfCycDesign [15]. The model exhibits a kalata-type cyclotide fold, with an antiparallel β-sheet and an overall well-defined structure (Fig. 4E). Prediction confidence (LDDT > 80) was reduced for loop 6, consistent with its unique sequence (Figure S8). Predicted aligned error (PAE) was low for the CCK-stabilized structure. Given the conserved sequence motifs in kalata-type cyclotides and the reported inhibition profile, these data strongly suggest that loop 3 of psysol 3 constitutes the primary binding site for human POP.

### Inhibitor psysol 3 protects oxytocin from degradation by human POP

**Fig. 5:**
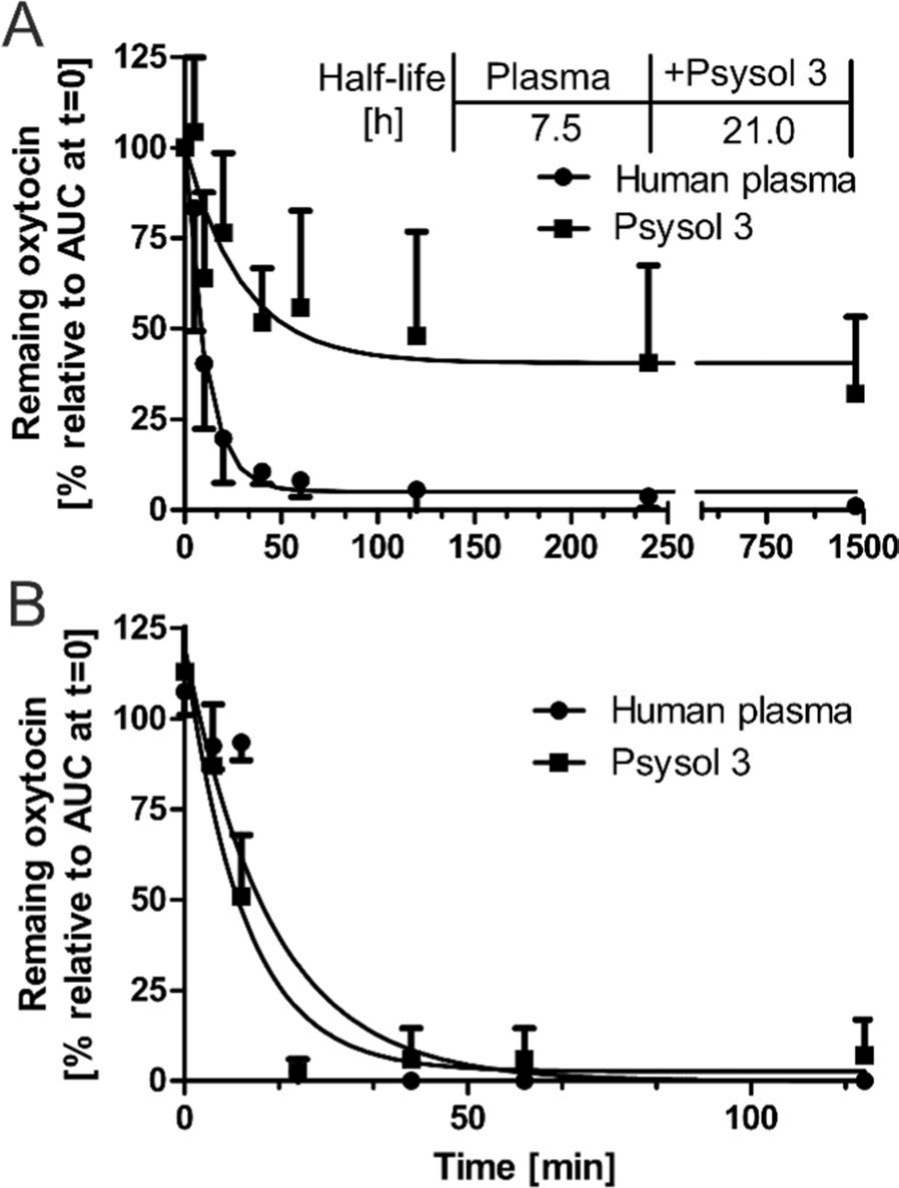
Psysol 3 inhibits the degradation of neuropeptide by POP. **A** Human OT (50 µM) and recombinant human POP (50 ng/mL) were co-incubated in protease assay buffer at 37 °C for 24 h. Sample aliquots were withdrawn at time points: 0, 5, 10, 20, 40, 60 and 120 min, as well as at 4 and 24 h. The remaining intact OT was analyzed by LC–MS analysis (*m/z* 504.2, [M + 2H]^2+^) and calculated to the timepoint zero. Under these conditions, the half-life of the analyte was circa 7.5 h, whereas in the presence of psysol 3 (13.5 µM, ~ tenfold IC_50_), the apparent half-life shifted to more than 21 h. This indicates an almost threefold increase in protease lytic resistance through the presence of the peptide inhibitor. The data are shown as n = 3 with mean ± SD. **B** A similar experiment incubating OT and psysol 3 together in active human plasma was conducted. The half-life of OT was determined to be about 10 min, whereas the addition of psysol 3 had no significant change in plasma half-life. The data are shown as n = 2 with mean ± SD

Cyclotides were first discovered in the 1970s, when a Norwegian physician observed the ethnomedicinal use of *kalata-kalata* (*Oldenlandia affinis L*.) via oral or intrauterine infusions [39]. Meanwhile, POP has been identified as an OT-degrading enzyme with significant expression in the placenta [40] and uterus [24]. Under physiological conditions, OT circulates at very low levels (1–10 pM) [41], reaching transient peaks during labor to induce myometrial contractions. To test whether psysol 3, the most potent cyclotide POP inhibitor reported to date, could affect OT degradation, it was incubated with recombinant human POP for 24 h and residual peptide was quantified by LC–MS. OT exhibited a half-life of τ = 6.7 h under these conditions, which increased to τ = 23.4 h in the presence of psysol 3 (13.5 µM; approx. tenfold its IC_50_) (Fig. 5A), demonstrating effective protection from POP-specific cleavage. In contrast, when OT degradation was determined in human plasma (where it is exposed to a broad spectrum of OT-degrading enzymes), psysol 3 did not alter degradation kinetics compared to the control (Fig. 5B). This suggests that the high activity of plasma proteases overrides the protective effect of psysol 3 under these conditions.

## Discussion

The genus Psychotria (Rubiaceae) is a large group with ~ 1600 species. Tropical *Psychotria spp.* are a rich source of natural products, including psychoactive *(P. viridis)* [42], antiparasitic *(P. klugii)* [43] and antimicrobial [44] alkaloids. In addition, numerous peptides have been reported, displaying diverse bioactivities, such as insecticidal [45], anti-cancer [46] and anti-proliferative activities against immune cells [38]. Our laboratory previously investigated peptide distribution in Psychotria [28], and several additional studies confirmed peptides in selected species [26, 38, 45–48]. Yet, the diversity of natural peptides across this large genus remains insufficiently characterized, warranting further exploration.

Here, we examined the peptidome of 11 Psychotria species. Using a peptide discovery workflow combining peptide-enriched extracts with MS [2], we screened samples with a fluorescent POP inhibition assay [26, 29]. All samples showed inhibition activity (IC_50_ of 27–208 µg/mL), consistent with previous reports [26, 29]. Given that ribosomally synthesized and post-translationally modified peptide (RiPP) families are often encoded in multiple precursor genes, plants can express extensive peptide libraries with varied sequences [3]. We found that peptide-containing samples exhibited higher inhibitory potency than those without detectable peptides. While POP substrates are typically small peptides (< 3 kDa), certain small molecules such as berberine [49] and oligo-O-galloyl glucoses [50] also display inhibitory activity. To validate our hypothesis, isolation of a peptide to purity was necessary.

Peptidomics analysis revealed the presence of the CCK motif in these Psychotria peptides, a defining feature of cyclotides. Among all species, *P. solitudinum* appeared the most interesting, with 37 peptides and strong inhibition activity. Using bioassay-guided isolation and reversed-phase chromatography [51], we isolated psysol 3 as the active principle. Its sequence was unique compared to entries in CyBase (www.cybase.au.org; accessed at 20.05.2025). Psysol 3 had low micromolar inhibition in line with typical peptide inhibitors [26, 51, 52] and slightly superior to previously reported cyclotide POP inhibitors (see Fig. 4B). Importantly, psysol 3 was not degraded by human POP, despite cyclotides being close to the substrate size threshold (~ 3000 Da) [53]. Sequence-guided synthesis of small cyclic probes highlighted proline-containing motifs as determinants of POP inhibition, suggesting catalytic-site targeting. Whether the full cyclotide accesses the catalytic pocket or binds secondary sites remains unresolved [54, 55].

A postulated model [22] suggests POP substrate entry requires major conformational changes, involving loop regions A (residues 189–209), B (residues 557–608), and C (residues 636–646) (numbers are based on the UniProtKB entry P48147) [54]. Only crystal structures of mammalian POP in closed conformations are available, limiting mechanistic insights [54, 56, 57]. Hence, rational design of POP inhibitors remains challenging [58]. To date, medicinal chemistry has produced potent peptidomimetic [59] and non-peptide inhibitors [60], including clinical candidates S17092 and JTP-4819, both discontinued [61]. A new strategy focuses on modulating POP’s non-catalytic protein–protein interactions (PPI), e.g., with α-synuclein or PP2A [55]. Recently, HUP-like 5-aminothiazoles selectively targeted a secondary His-loop site [55, 62]. Given that peptides are highly effective PPI modulators [63], natural or synthetic peptide libraries combined with high-throughput POP assays offer promising opportunities for future discovery.

We further assessed whether psysol 3 affected endogenous POP substrates, using OT as a model. POP normally inactivates OT via C-terminal cleavage. In vitro, psysol 3 reduced OT degradation, but no effect was observed in human plasma, where oxytocinases rapidly degrade the hormone. While clinical applications of OT analogs (e.g., carbetocin) remain limited to postpartum hemorrhage, selective peptide inhibitors may overcome challenges faced by small-molecule ligands. This concept is supported by hirudin-derived anticoagulants, which target thrombin exosites with exceptional potency and selectivity [11]. Similarly, engineering of the squash-derived cyclotide MCoTI-II yielded cMCoFx1, a highly selective thrombin inhibitor [64, 65]. These examples underscore the therapeutic promise of stabilized peptide scaffolds such as cyclotides.

More broadly, peptide natural products have repeatedly served as blueprints for first-in-class drugs [5, 9, 12, 66]. A landmark example is the development of ACE inhibitors from the bradykinin-potentiating peptide of the venomous pit viper (*Bothrops jararaca*) [67], which inspired the approved drugs captopril, lisinopril, and enalapril. Likewise, venom-derived RGD mimetics underlie the antiplatelet drugs tirofiban and eptifibatide [5, 7]. These precedents emphasize the translational potential of natural peptides in drug discovery.

## Conclusions

With recent advances in AI and cheminformatics, peptides have become increasingly amenable to virtual and combinatorial screening, accelerating protease-targeted drug development [63]. This study identified psysol 3, a novel macrocyclic inhibitor of human POP with unique sequence features and robust stability. Its CCK framework confers resistance to proteolysis and potential cell permeability, making psysol 3 a valuable probe for investigating POP biology and a promising starting point for therapeutic development.

## Supplementary Information

Additional file 1.

## Data Availability

Data supporting the findings of this study is available in the supplementary online material. Annotated peptide genes of *P. solitudinum* have been deposited at www.cybase.au.org.
