# Supplementary material for: Tropical psychotria plants are a rich source for peptide inhibitors of human prolyl oligopeptidase
Source: Nat Prod Bioprospect. 2026 Apr 14;16(1):54. doi: 10.1007/s13659-026-00598-z (PMC13076805; doi:10.1007/s13659-026-00598-z)

# Supplementary Information

# Tropical Psychotria Plants are a Rich Source for Peptide Inhibitors of Human Prolyl Oligopeptidase

Roland Hellinger^1^, Paula Schwarz^1^, Jonathan Dieringer^1^, Carina Ebermann^1^, Kirtikumar B. Jadhav^2^, Markus Muttenthaler^2,3^ and Christian W. Gruber^1,*^

^1^ Center for Pharmacology and Physiology, Medical University of Vienna, 1090 Vienna, Austria

^2^ Institute of Biological Chemistry, Faculty of Chemistry, University of Vienna, 1090 Vienna, Austria

^3^ Institute for Molecular Bioscience, The University of Queensland, 4072 Queensland, Australia.

*Correspondence: Christian W. Gruber, christian.w.gruber@meduniwien.ac.at, Tel. +43 40160 31390

# Supplementary Data

## Supplementary Data S1.

Annotation notes: The start/stop codons have been marked in black within the scaffold cDNA
sequences, where applicable; ER signals have been assigned by similarity to known cyclotide precursors (underlined); the mature cyclotide sequences have been marked in grey in the translated protein
sequences; where applicable, the stop codon in the translated sequence has been indicated by an asterisk in the protein sequence.

**>TRINITY_DN1006_c1_g1_i2 len=461 path=[0]**

TTCTAAGGCAGACAAAATATGGCCGCAGCTGCATCCAGGAGTATTGCATACCCCACTAAAACATGTTTCACCACAAATTGGTAATCCACGAACCATCTCACTTCCCAAATTGGCACCGTCCAGTCTGTCATAAGAGTCTACTTCCAAGGTCATAAGCGAGGCAACGAGGAAAGAGAGGAAGAGGAGAGTAGCAACCTTAGCCATTGGATCACTTGGCAGAATTTGTTGAAGGAATTAAAATTAAGCTCCCAAAGGAATGAATAGTTCTGCTGA

Reverse complement

TCAGCAGAACTATTCATTCCTTTGGGAGCTTAATTTTAATTCCTTCAACAAATTCTGCCAAGTGATCCAATGGCTAAGGTTGCTACTCTCCTCTTCCTCTCTTTCCTCGTTGCCTCGCTTATGACCTTGGAAGTAGACTCTTATGACAGACTGGACGGTGCCAATTTGGGAAGTGAGATGGTTCGTGGATTACCAATTTGTGGTGAAACATGTTTTAGTGGGGTATGCAATACTCCTGGATGCAGCTGCGGCCATATTTTGTCTGCCTTAGAA

3'5' Frame 1

SAELFIPLGA-F-
FLQQILPSDPMAKVATLLFLSFLVASLMTLEVDSYDRLDGANLGSEMVRGLPICGETCFSGVCNTPGCSCGHILSALE

**>TRINITY_DN106_c1_g1_i2 len=461 path=[0]**

ACAGATCGGTAGCCCACCCACCATCTCGCTTCCCAGAGTAGCTACTTCCGGTGTTAGTCCATTTTTCGTGCATATAGGATATGAACAACTGCAACCAGGGGTATTACATGTGCCAGTGAAGCATGTTTCACCACAGATCGGTAGCCCACCCACCATCTCGCTTCCCAGAGTAGCTACTTCCGGTGTTAGTCCATTTTTCGTGCATATAGGATATGAACAAGTGCAACCAGGGGTATAACATTTTGTAGTGAAGCATGTTTCACCACAGATCGGTAGCCCACCCACCAGCTCGCTTCCCAAATGGGGGACTTGCATTCTGTCGTAAGATTCCACTTTCAGCATCACAATCGCGGCAATGAGGAACAAGAGGAAAGGTGAGCATAAAATTTTGCCATTTGATCACTTGGCAGGATTTTTTGAAGGAACTATAAGCTGGCTGCAGGAATTAATATGTTGCTTGG

Reverse complement

ccaagcaacatattaattcctgcagccagcttatagttccttcaaaaaatcctgccaagtgatcaaatggcaaaattttatgctcacctttcctcttgttcctcattgccgcgattgtgatgctgaaagtggaatcttacgacagaatgcaagtcccccatttgggaagcgagctggtgggtgggctaccgatctgtggtgaaacatgcttcactacaaaatgttatacccctggttgcacttgttcatatcctatatgcacgaaaaatggactaacaccggaagtagctactctgggaagcgagatggtgggtgggctaccgatctgtggtgaaacatgcttcactggcacatgtaatacccctggttgcagttgttcatatcctatatgcacgaaaaatggactaacaccggaagtagctactctgggaagcgagatggtgggtgggctaccgatctgt

3'5' Frame 3

KQHINSCSQLIVPSKNPAKCSNGKILCSPFLLFLIAAIVMLKVESYDRMQVPHLGSELVGGLPICGETCFTTKCYTPGCTCSYPICTKNGLTPEVATLGSEMVGGLPICGETCFTGTCNTPGCSCSYPICTKNGLTPEVATLGSEMVGGLPIC

**>TRINITY_DN97_c0_g1_i2 len=460 path=[0]**

GGAGCTTAATTATAATATTCGTTCAACAAATTCTCACAAGTGATATCAAATGGCTAAGTTTGCTAATCACCTCTTCCTCTTTTTCCTCATTGCCTCGGTTGTGATGTTGGAAGTGAATTCCTACGACAGAATCCAAGTCCCCAATTTGGCAGAGGAGCTAGTTGGTGGTAGCATAGTGGCCTGTGGTGAATCGTGCGTCACGGGGACGTGCTATACTCCGGGTTGCACTTGCTCTTGGCCTGTTTGCAAAAAGAATGGACTAAATCCTGAGAGTCTTTAATGTGGTTGACGGTGTATTAATCTTTTTAGTACGATCTAAATTATGGAACTATTTATTGTGATCTTAATGTAATGTAAGCTTGTTTATGTTCTGCATGGTTTGGCTTGGTTTGGTGTGTTTTCTGCGTCTGTCTAAATAAAGTCTTTTGTCTTGTTGAATTATGTTCAATTAAAATACGAC

5'3' Frame 2

ELNYNIRSTNSHK-YQMAKFANHLFLFFLIASVVMLEVNSYDRIQVPNLAEELVGGSIVACGESCVTGTCYTPGCTCSWPVCKKNGLNPESL*-CG-RCINLFSTI-IMELFIVILM-CKLVYVLHGLAWFGVFSASV-IKSFVLLNYVQLKYD

**>TRINITY_DN97_c0_g1_i1 len=269 path=[0]**GGAGCTTAATTATAATATTCGTTCAACAAATTCTCACAAGTGATATCAAATGGCTAAGTTTGCTAATCACCTCTTCCTCTTTTTCCTCATTGCCTCGGTTGTGATGTTGGAAGTGAATTCCTACGACAGAATCCAAGTCCCCAATTTGGCAGACGAGCTAGTTGGTGGTAGCGCAACGGCCTGTTTTGAATCGTGCGTCAAGGGGAAGTGCTATACTCCTGGTTGCACTTGCTATTGGCCTGTTTGCAAAAGAATGGACTAATCTCGAG

5'3' Frame 2

ELNYNIRSTNSHK-YQMAKFANHLFLFFLIASVVMLEVNSYDRIQVPNLADELVGGSATACFESCVKGKCYTPGCTCYWPVCKRMD*-SR

>contig00008 length=585 numreads=244

CAAGCAACATATTAATTCCTGCAGCCAGCTTATAGTTCCTTCAAAAAATCCTGCCAAGTGATCAAATGGCAAAATTTTcTGCTCACCTTTCCTCTTGTTCCTCATTGCCGCGATTGTGATGCTGAAAGTGGAATCTTACGACAGAATGCAAGTCCCCCATTTGGgAAGCGAGCTGGTGGGTGGGCTACCGAcCTGTGGTGAAACATGCTTCACTACAAAATGTTATACCCcTGGTTGCtCTTGTTCATATCCTaTATGCacGAAAAaTGGACTAAcACCGGAAGTAgCTACTCTGGGAAGCGAGATGGTGGGTGGGCTACCGATCTGTGGTGAAACATGCTTCACTGGCAaATGTAATACCCCTGGTTGCAGTTGTTCATATCCTATATGCACGAAAAaTGGACTGAATCCTGAAAATATGTAAGATGGTGGACGGTGTGCTACTCTTTtCAATGCCATCTTAAGAATAAAAGGATCCAATGAGATCTTAATGTATGTTtAAGTAAaTTTccTTTTTTttGCATGATTTGGTTTGTTTTGTGAAGCGTGCTTTTtGTTTTTtGTCTTTGTGaTTATcTTCAATTC

5'3' Frame 2

KQHINSCSQLIVPSKNPAK-SNGKIFCSPFLLFLIAAIVMLKVESYDRMQVPHLGSELVGGLPTCGETCFTTKCYTPGCSCSYPICTKNGLTPEVATLGSEMVGGLPICGETCFTGKCNTPGCSCSYPICTKNGLNPENM*-DGGRCATLFNAILRIKGSNEILMYV-VNFLFFA-FGLFCEACFLFFVFVIIFN

## Supplementary Data S2.

Sequence similarity and identity analysis from reported cyclotide peptide sequences. The primary sequence of psysol 3 was searched towards MEROPS inhibitors and SwissProt/UniprotKB databases. The top 26 hits were reported applying the threshold E^0.00016^.

**Peptide Sequence Number of Residues Similarity Identity**

psysol 3 -GLPTCFETCILGTCYTPGCSCSTYRLCLNN 30

kalata_B9 GSVFNCGETCVLGTCYTPGCTCNTYRVCTKD 31 92.3 69.2

mela_7 -GLPTCGETCFKGKCYTPGCSCS-YPICKKN 29 80.0 70.0

kalata_B6 -GLPTCGETCFGGTCNTPGCSCSSWPICTRN 30 80.0 66.7

kalata_B3 -GLPTCGETCFGGTCNTPGCTCDPWPICTRD 30 80.0 66.7

kalata_B10 -GLPTCGETCFGGTCNTPGCSCSSWPICTRD 30 85.2 70.4

Cliotide_T18 -GLPICGETCFTGTCYTPGCTCS-YPVCKKN 29 80.0 66.7

mela_6 -GIPTCGETCFKGKCYTPGCSCS-YPICKKD 29 81.5 74.1

cycloviolacin_O14 GSIPACGESCFKGKCYTPGCSCSKYPLCAKN 31 79.3 62.1

vibi_C -GLPVCGETCAFGSCYTPGCSCS-WPVCTRN 29 80.0 63.3

cycloviolacin_O21 -GLPVCGETCVTGSCYTPGCTCS-WPVCTRN 29 80.0 60.0

Cter_M -GLPTCGETCTLGTCYVPDCSCS-WPICMKN 29 83.3 66.7

cycloviolacin_O24 -GLPTCGETCFGGTCNTPGCTCDPWPVCTHN 30 80.0 60.0

mden_A -GIPTCGETCTLGTCNTPGCTCS-WPICTKN 29 80.0 63.3

mden_B -GLPICGETCFTGKCYTPGCTCS-YPICKKN 29 76.6 63.3

varv_peptide_H -GLPVCGETCFGGTCNTPGCSCETWPVCSRN 30 76.6 63.3

vibi_B -GLPVCGETCFGGTCNTPGCTCS-YPICTRN 29 76.6 66.7

Mobo_A -GFPTCGETCTLGTCNTPGCTCS-WPICTRN 29 76.6 63.3

varv_peptide_B -GLPVCGETCFGGTCNTPGCSCDPWPMCSRN 30 76.6 63.3

mela_2 -GKPTCGETCFKGKCYTPGCTCS-YPLCKKD 29 77.8 70.4

psyleio_A -GLPICGETCFTGTCNTPGCSCT-YPICTRD 29 77.8 70.4

vibi_A -GLPVCGETCFGGTCNTPGCSCS-YPICTRN 29 76.7 63.3

kalata_S -GLPVCGETCVGGTCNTPGCSCS-WPVCTRN 29 76.7 63.3

CyCloviolacin_O12 -GLPICGETCVGGTCNTPGCSCS-WPVCTRN 29 73.3 63.3

VinC_A -GIPVCGETCTLGTCYTAGCSCS-WPVCTRN 29 76.6 63.3

kalata_B8 GSVLNCGETCLLGTCYTTGCTCNKYRVCTKD 31 84.6 61.5

mang_A -GFPTCGETCTLGTCNTPGCTCS-WPICTRD 29 81.5 66.7

* *:* * .* * * :

# Supplementary Tables

## Table S1. Peptidomic analysis of *P. solitudinum* C_18_ extract sample

| Peptide ID | MS1 fingerprint[*m/z*]^1^ | Transcriptome^2^ |
| --- | --- | --- |
| Psysol 13 | 2869.2 | n.d. |
| Psysol 14 | 2881.2 | n.d. |
| Psysol 15 | 2890.7 | n.d. |
| Psysol 2 | 2905.2 | n.d. |
| Psysol 16 | 2921.6 | n.d. |
| Psysol 17 | 2926.8 | n.d. |
| Psysol 7 | 2946.3 | full |
| Psysol 18 | 2967.9 | n.d. |
| Psysol 19 | 2972.0 | n.d. |
| Psysol 20 | 3006.0 | n.d. |
| Psysol 21 | 3020.1 | n.d. |
| Psysol 9 | 3029.0 | full |
| Psysol 22 | 3030.0 | n.d. |
| Psysol 23 | 3037.3 | n.d. |
| Psysol 24 | 3050.4 | n.d. |
| Psysol 25 | 3062.9 | n.d. |
| Psysol 26 | 3073.3 | n.d. |
| Psysol 27 | 3078.3 | n.d. |
| Psysol 6 | 3079.2 | full |
| Psysol 8 | 3086.0 | full |
| Psysol 1 | 3089.4 | partial |
| Psysol 28 | 3101.1 | n.d. |
| Psysol 29 | 3108.0 | n.d. |
| Psysol 12 | 3111.2 | full |
| Psysol 5 | 3136.3 | full |
| Psysol 30 | 3140.4 | n.d. |
| Psysol 31 | 3146.4 | n.d. |
| Psysol 32 | 3168.1 | n.d. |
| Psysol 3 | 3209.5 | full |
| Psysol 11 | 3224.2 | full |
| Psysol 33 | 3274.2 | n.d. |
| Psysol 34 | 3313.0 | n.d. |
| Psysol 35 | 3324.4 | n.d. |
| Psysol 36 | 3360.7 | n.d. |
| Psysol 37 | 3376.6 | n.d. |
| Psysol 38 | 3417.4 | n.d. |
| Psysol 39 | 3447.2 | n.d. |
| Psysol 40 | 3448.3 | n.d. |
| ^1^ Experimental mass signal detected with mass accuracy ± 100 ppm  ^2^ Partial characterizes a partial precursor sequence with a full mature peptide sequence; Full = full precursor sequence identified.  n.d. not detected | | |

## Table S2. Yields of preparative HPLC fractionation and counted peptide signals per fraction.

| Fraction # | Amount Yield [mg]^a^ | Number of putative peptide *m/z* mass signals |
| --- | --- | --- |
| 1 to 10 | >2 mg^b^ | 2 |
| 9 | 12.7 | 1 |
| 10 | 14.7 | 2 |
| 11 | 19.4 | 14 |
| 12 | 13.5 | 15 |
| 13 | 12.5 | 23 |
| 14 | 9.4 | 23 |
| 15 | 5 | 12 |
| 16 | 4.8 | 10 |
| 17 | 2.8 | 14 |
| 18 | 1.1 | 4 |
| 19 | 1.4 | 5 |
| 20 | 0.8 | 0 |
| Sum | **98.1** | **n.a.** |
| ^a^ The numbers were received from a single representative preparative HPLC fractionation of *P.* *solitudinum* C_18_ extract sample (100 mg).  ^b^ The weights yield of fraction 1 to 10 was not determined. | | |

## Table S3. Amino Acid Analysis

| Amino acid^1^ | Mol% | Number of AA |
| --- | --- | --- |
| Ser | 7.7 | 2 |
| Arg | 4.1 | 1 |
| Gly | 13.7 | 3 |
| Asp/Asn^2^ | 8.9 | 2 |
| Glu/Gln^2^ | 5 | 1 |
| Thr | 19.2 | 5 |
| Pro | 8.7 | 2 |
| Tyr | 7.5 | 2 |
| Ile | 4.3 | 1 |
| Leu | 16.5 | 4 |
| Phe | 4.4 | 1 |
| Met^3^ | n.d. | 0 |
| Val^3^ | n.d. | 0 |
| His^3^ | n.d. | 0 |
| Ala^3^ | n.d. | 0 |
| ^1^ Cys and Trp residues were not detectable in the amino acid analysis, since acid hydrolysis damages these amino acids.  ^2^ Acid hydrolysis converts Gln to Glu and Asn to Asp. Hence, the reported of Glu residues are a sum of Gln+Glu. The reported Asp residues are a sum of Asn+Asp  ^3^ Met, Lys, Val, Ala & His were not detected in the peptide.  n.d.: these amino acids were not detectable. | | |

# Supplementary Figures

## Figure S1. Screening of plant extracts from the Psychotria tribe toward inhibition of PREP.

**A.** In total, 11 Psychotria plant extracts, *P. polypheblia, -solitudinum, -macrophylla, -capitata, C. ipecacuanha, -poeppigiana, -pilosa, -erecta, -mortoniana, -borucana,* and *-elata*, were screened for inhibition of PREP activity. From each sample 50, 100, 200 and 400 µg/mL were evaluated for the inhibition activity of peptide-enriched plant extracts. The inhibitory activity (%) was calculated as (1 – remaining activity relative to the enzyme activity) x 100. The specific PREP inhibitor Kyp-2047 was used in 1 µM as a positive control. The data show three independent experiments with ± standard deviation **B.** The obtained data were fitted to a non-linear regression with constraints; bottom and top are equal to 0 and 100%, respectively. **C.** The curve fit enabled us to determine the concentrations for half inhibitory activity (IC_50_), which are provided in the table.

**
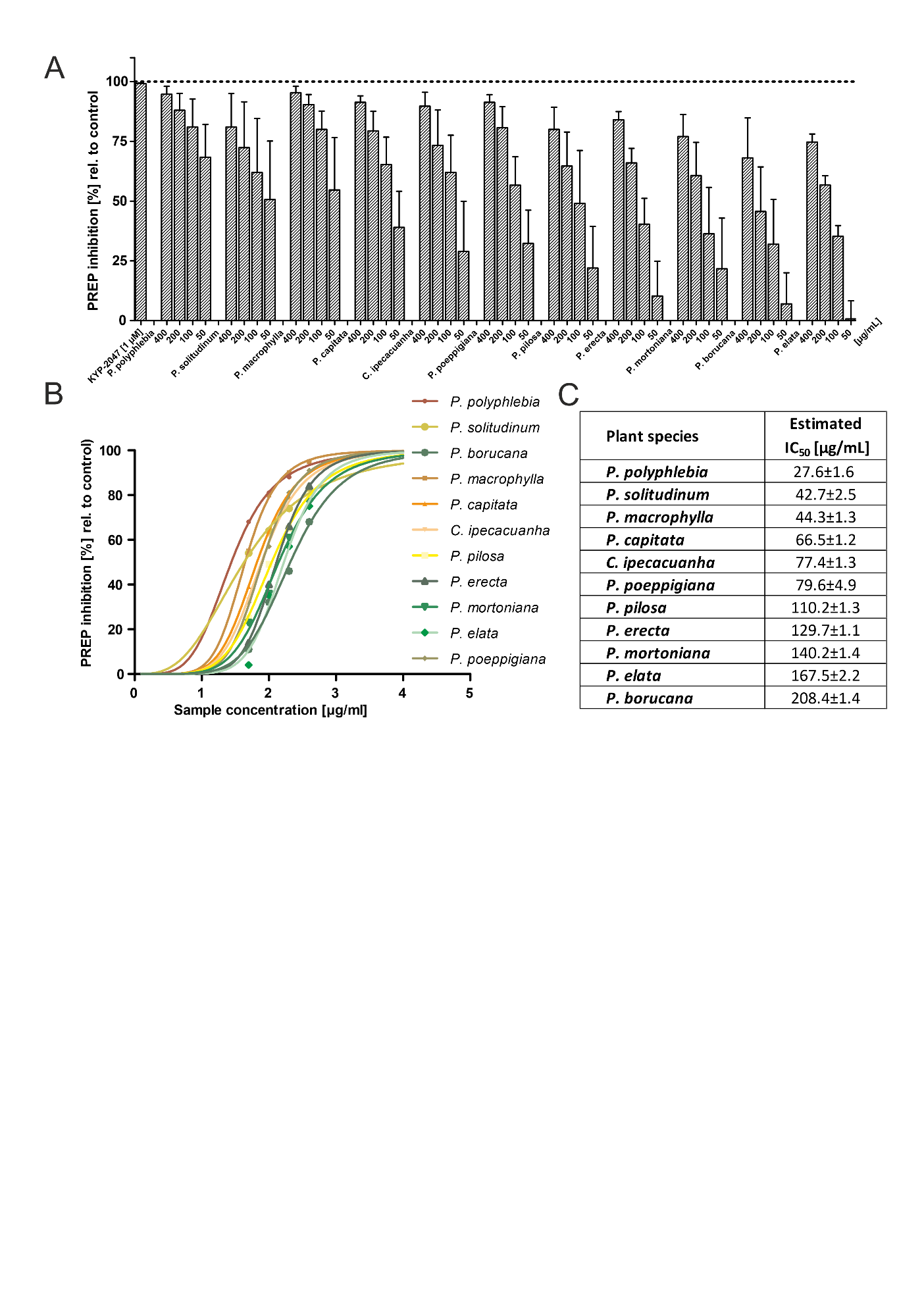
**

## Figure S2. MALDI-MS analysis of plant extracts from the Psychotria tribe for cyclotides

Mass spectra showing representative data for 11 Psychotria species. **A.** Peptide-enriched samples were analyzed *via* MALDI-TOF-MS, LC-MS and transcriptome mining. Mass signals in the range of *m/z* 2000-4000 were counted as peptide signals, as expected for cyclotide or knottin-type molecules. A correlation was tested between peptide counts, ranging from approximately 50 to only 2 counted molecules per plant sample, and POP inhibition activity (IC_50_). A coefficient of determination, R^2^ = 0.576, was found for the set of 11 plant samples. Mass spectral data for *P. polyphlebia* (**B.**), *P. solitudinum* (**C.**), *P.* *macrophylla* (**D.**), *P. capitata* (**E.**), *C.* *ipecacuanha* (**F.**), *P. poeppigiana* (**G.**), *P. pilosa* (**H.**), *P. erecta* (**I.**), *P. mortoniana* (**J.**), *P. borucana* (**K.**), and *P. elata* (**L.**) are provided as recorded by MALDI-MS-TOF analysis.


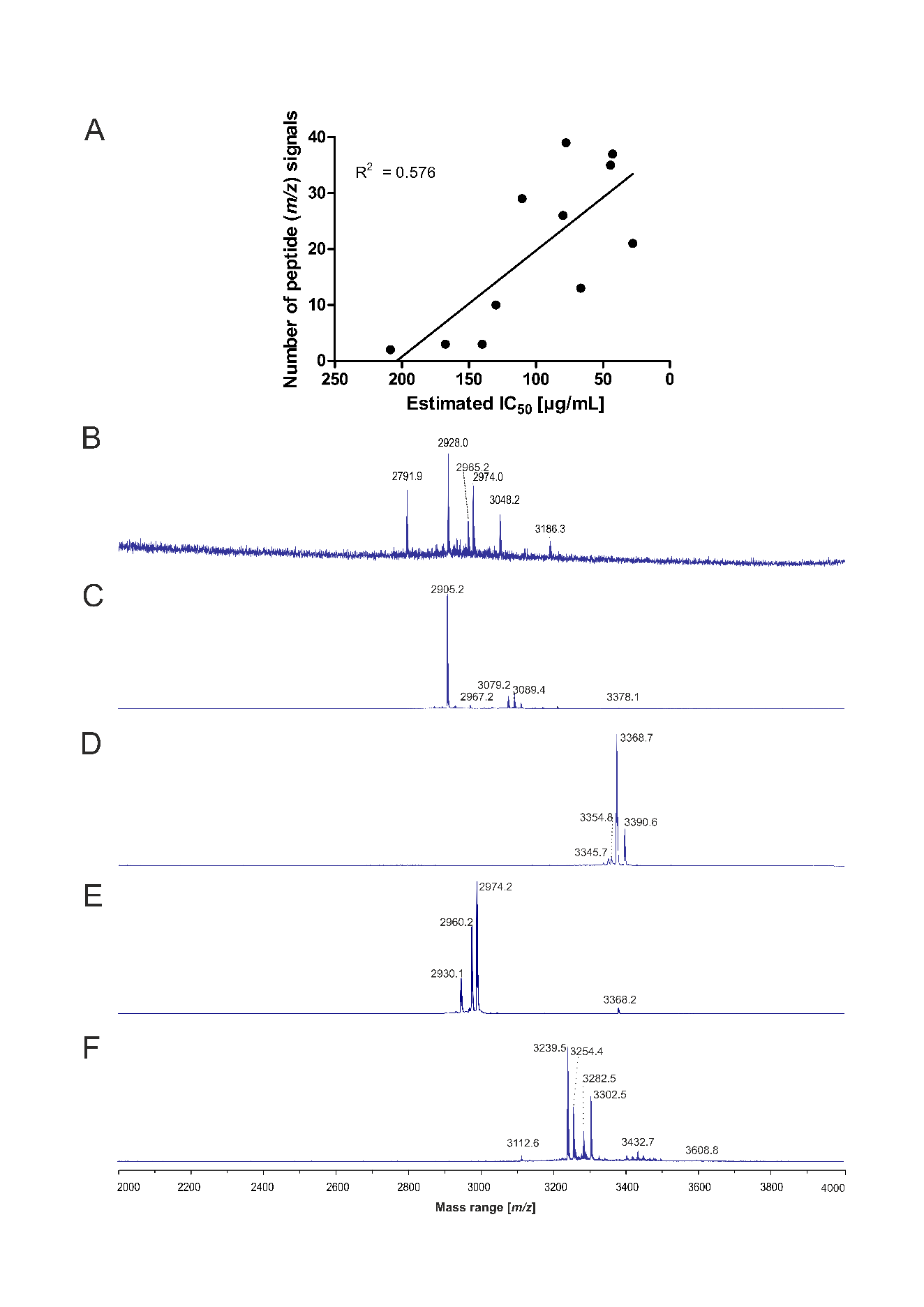

**
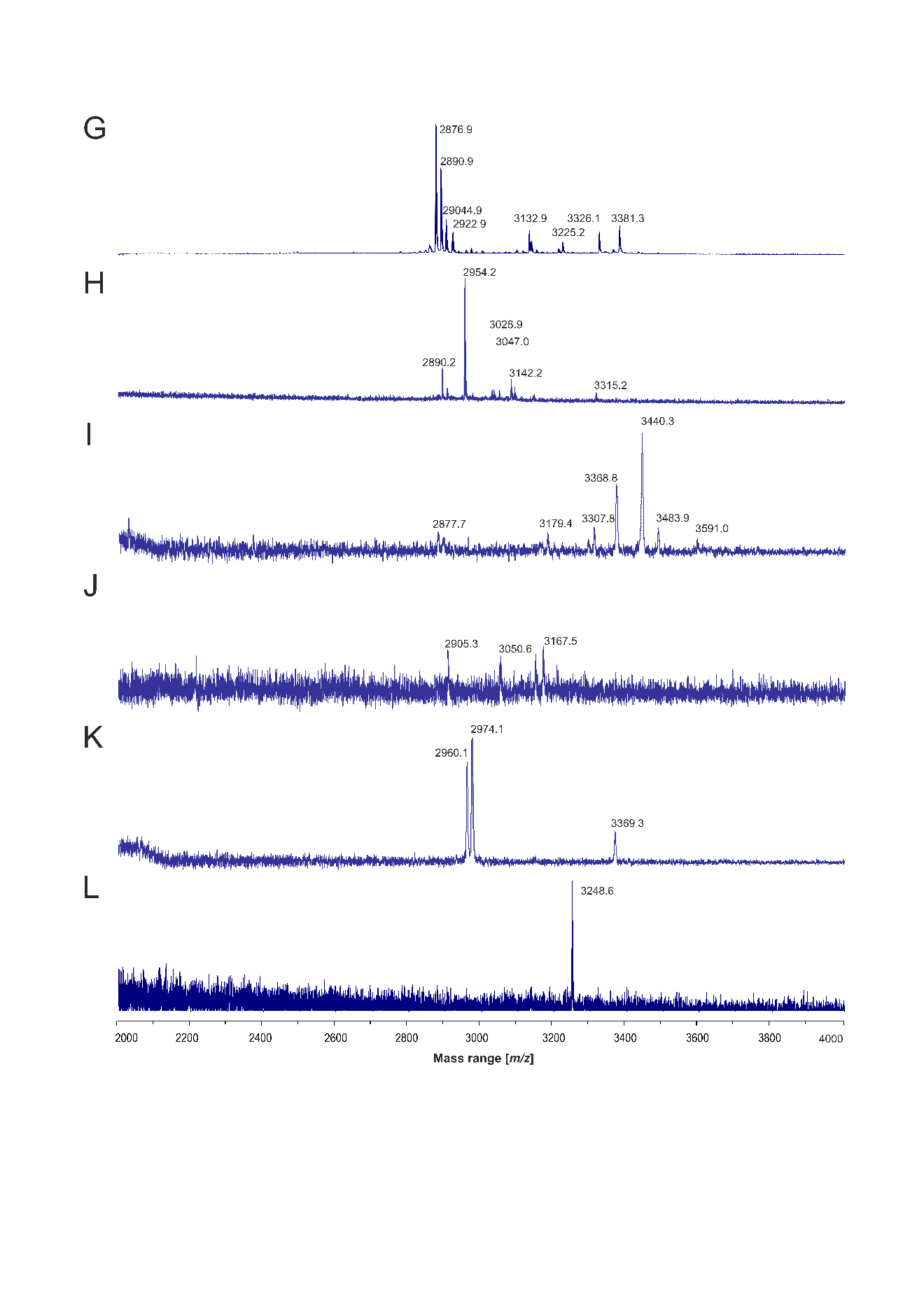
**

## **Figure S3. MALDI-MS analysis for the preparative HPLC fractions from *P. solitudinum*.**

The dried homogenized fraction material was analyzed with mass spectrometry to identify putative peptide mass signals in these samples. The mass range of *m/z* 2000 to 4000 is provided, and mass signals are labeled with a signal-to-noise level greater than 15. **A.** fraction (F)-1, **B.** F2, **C.** F3, **D.** F4, **E.** F5, **F.** F6, **G.** F7, **H.** F8, **J.** F9, **I.** F10, **K.** F11, **L.** F12, **M.** F13, **N.** F14, **O.** F15, **P.** F16, **Q.** F17, **R.** F18, **S.** F19, **T.** F20.

|  A |
| --- |
|  BA. |
|  CA. |
|  D |
| ****  E |
|  F |
| GA.  |
|  HA. |
|  I |
|  J |
|  K |
|  L |
|  MA. |
|  NA. |
|  OA. |
|  PA. |
|  QA. |
| **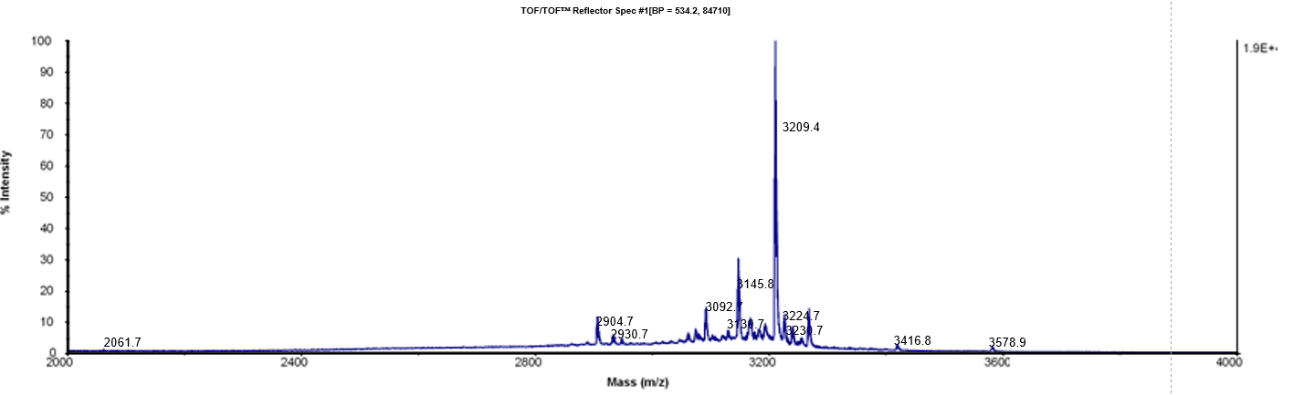**  R |
| **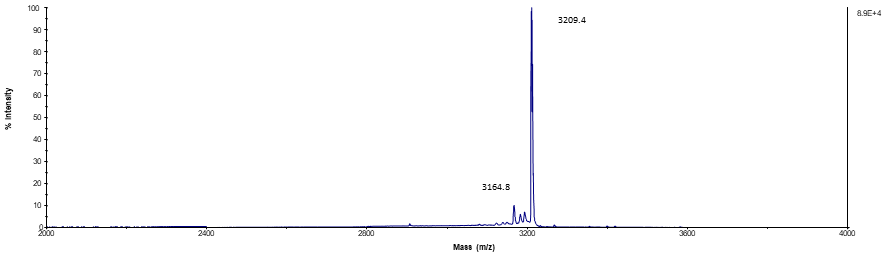**  S |
|  TA. |

## Figure S4. Trypsin and endoprotease GluC digestion experiment and *de novo* annotation of the GluC fragment.

The isolated peptide psysol 3 was treated with dithiothreitol and iodoacetamide to obtain a precursor peptide for proteolysis experiments. **A.** The proteolysis experiment with endoprotease GluC is shown. The digested sample was analyzed with MALDI-MS search for proteolytic fragments. A single resulting product with *m/z* 3575.4 was identified. **B.** The proteolysis experiment with trypsin is shown. The digestion experiments yielded a single fragment with *m/z* 3575.4. **C.** The GluC-derived fragment with *m/z* 3575.4 was applied in MS/MS fragmentation experiments on an autoflex speed MALDI-TOF/TOF-MS system. The precursor showed weak fragmentation behavior using CID fragmentation. The *de novo* fragmentation spectra annotation for the GluC-prepared precursor obtained partial sequence information; the conclusive full-length sequence information was obtained with further combined information (*e.g.,* the trypsin-digested precursor).

**
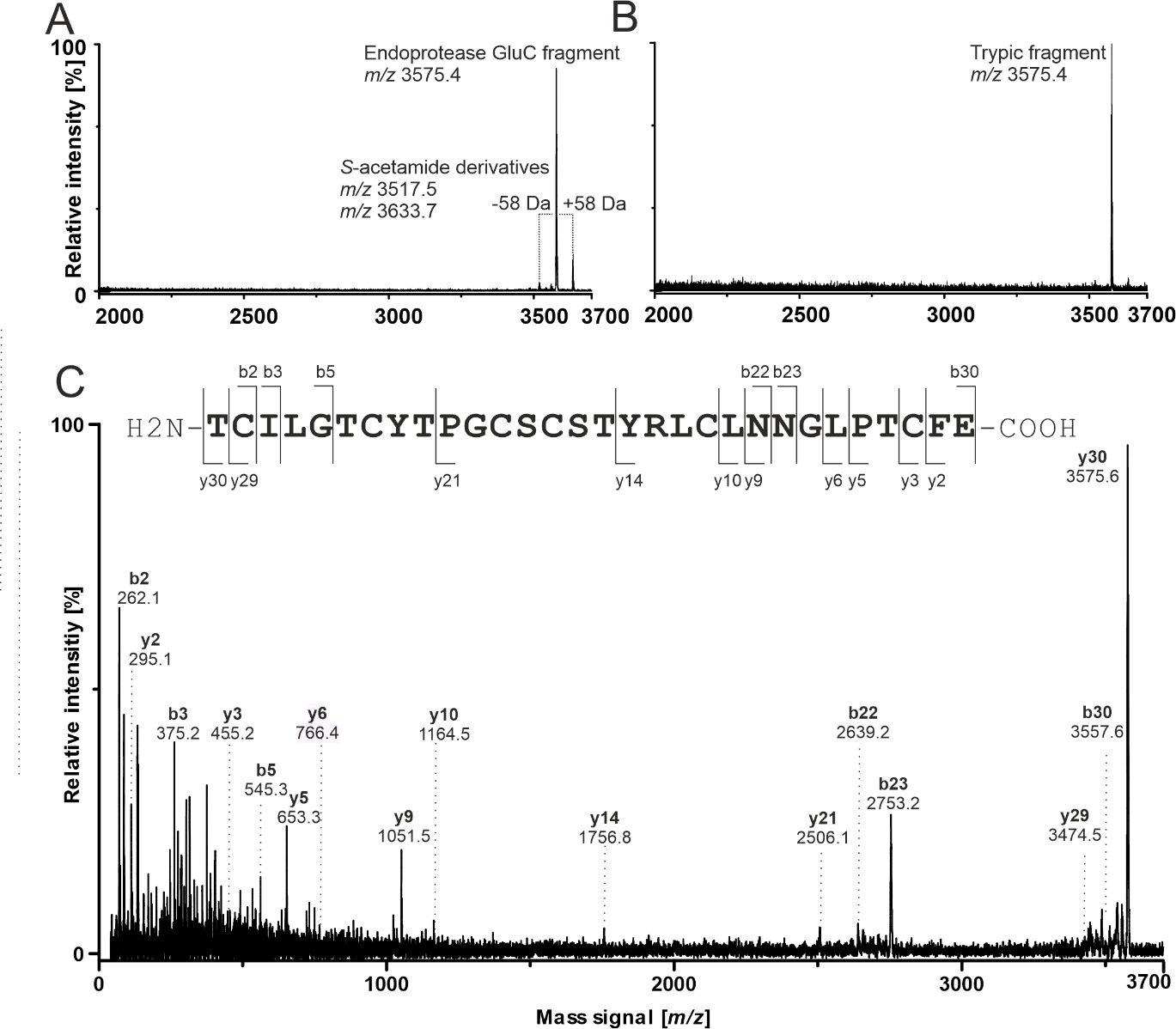
**

## Figure S5. Sequence logo of reported cyclotide inhibitors of human POP.

**A.** The determined sequence of psysol 3 was compared to all cyclotide entries published at [www.cybase.au.org](http://www.cybase.au.org) (>500) in an intercysteine loop-wise manner. The amino acid sequences of psysol 2, -3, kalata B1, -S and alca 1, -2 were used to prepare a residue frequency sequence logo (<https://weblogo.berkeley.edu/logo.cgi>). Amino acids with acid functionalities in the side chain are shown in red, and those with basic ones are shown in blue. The conserved cysteines are indicated in yellow, whereas the prolines are shown in green. **B.** The peptides psysol 2 and -3 were tested at 1, 10 and 100 µM toward the inhibition of human POP. The remaining enzyme activity was calculated from the slope of product formation, recorded via fluorescence, from a kinetic steady-state experiment. Three independent experiments were performed and the data show standard deviation. Student’s t-test was performed and indicated a significant difference for the 10 µM concentration level. (** P<0.01).

**
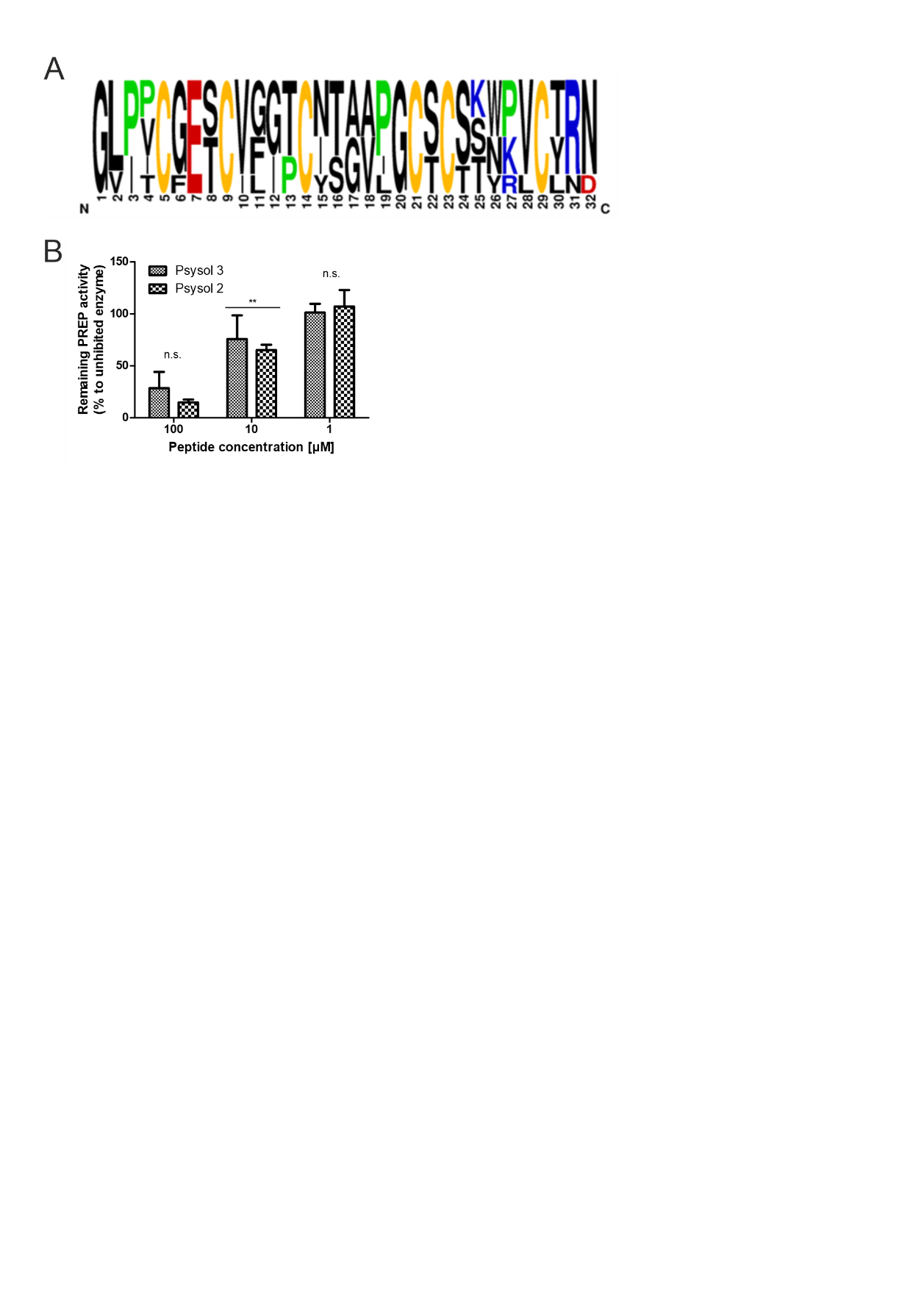
**

## Figure S6. Quality control analysis of synthetic peptide psysol 3

**A.** Psysol 3 was synthesized by a solid-phase peptide precursor, giving the fully protected linear peptide after the release from the resin. The cyclic peptide is obtained *via* standard peptide bond chemistry, and the peptide is oxidatively folded to give the native folded cyclic peptide. **B.** Native folded psysol 3 was purified to ≥95% purity, estimated by absorbance analyzing the A214 nm trace. **C.** The experimental mass obtained was *m/z* 3209.4. **D.-E.** Co-elution experiments were performed to test the native fold of the synthetic material in comparison to peptide material isolated from the plant. The synthetic material co-eluted nicely with the isolated peptide (R_t_ 26.7 min) as indicated by a mixed sample with spiked peptides 1:1 each.


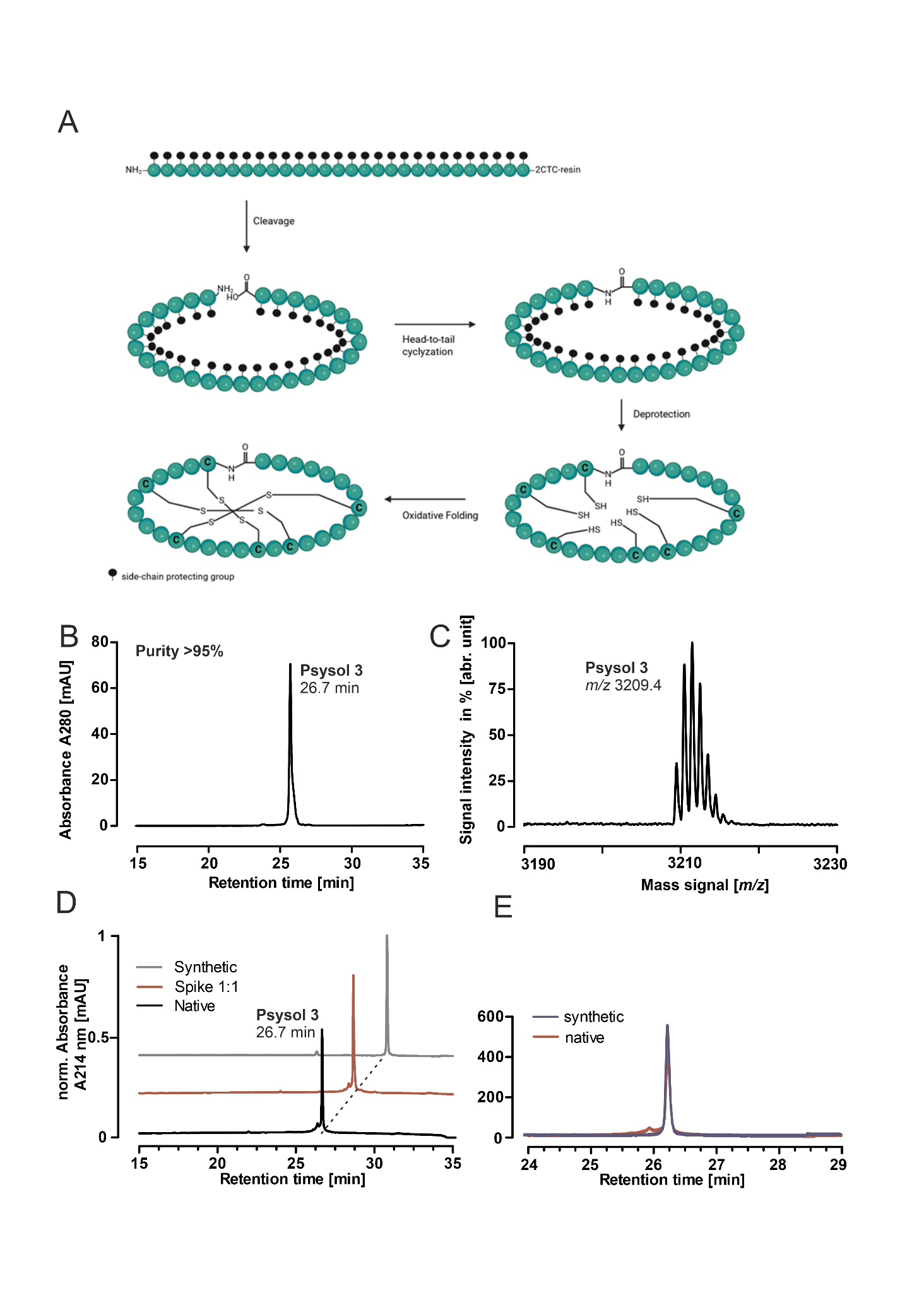


## **Figure S7. Quality control analysis of synthetic small disulfide cyclic peptide probes.**

**A–B.** Analytical RP-HPLC (**A**) and MALDI-TOF mass spectrum (**B**) of c(YTPG), showing a single product peak at 15.2 min and a monoisotopic [M+H]⁺ signal at *m/z* 582.4. **C–D.** Analytical RP-HPLC (**C**) and MALDI-TOF mass spectrum (**D**) of c(NGLPT), with a retention time of 15.0 min and a detected *m/z* of 646.4. **E–F.** Analytical RP-HPLC (**E**) and MALDI-TOF mass spectrum (**F**) of c(STYRL), with a retention time of 13.4 min and a monoisotopic mass of *m/z* 784.6. RP-HPLC chromatograms were recorded at an absorbance of 214 nm. All synthetic cyclic peptides were purified by preparative HPLC and obtained at >95% purity.


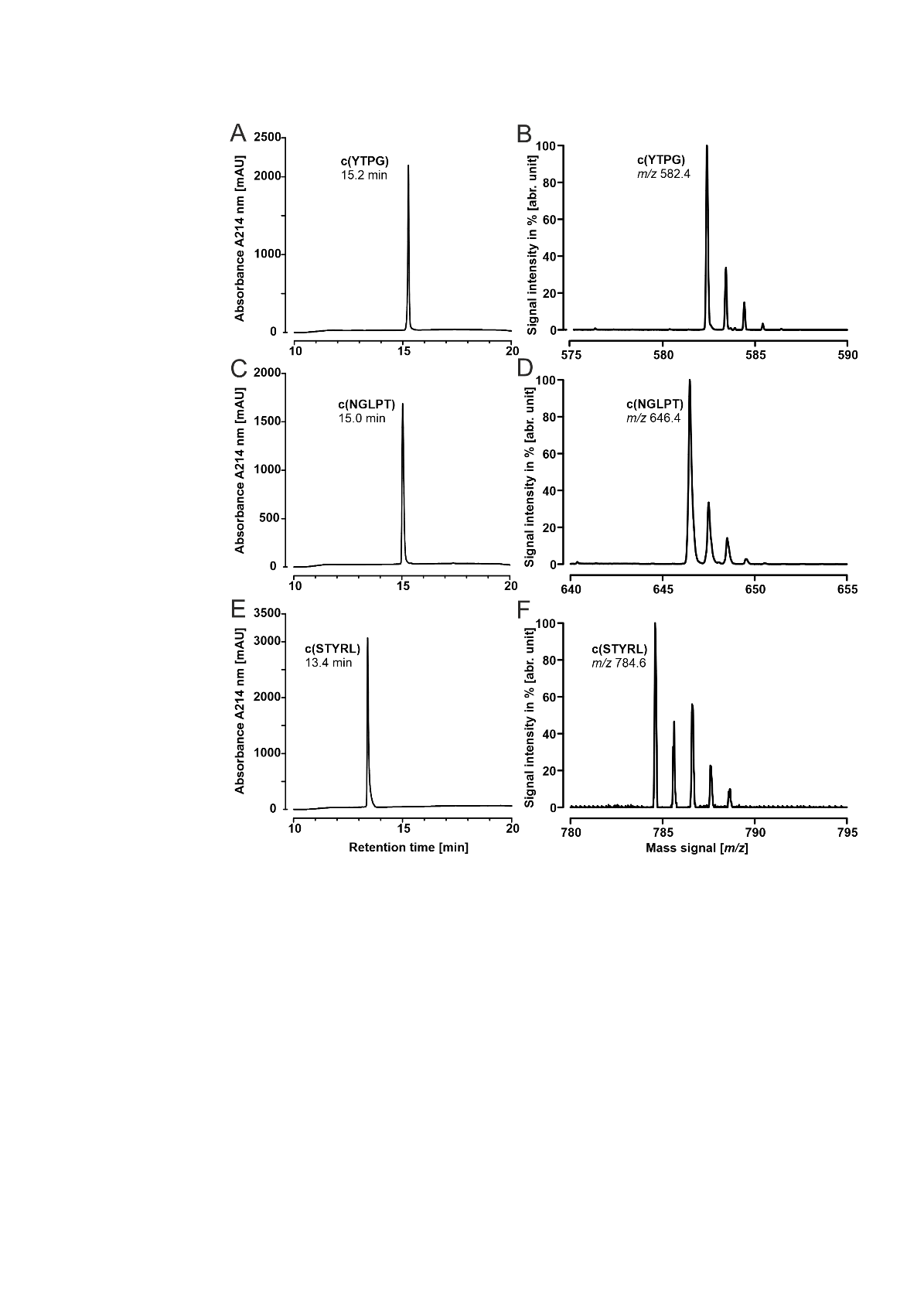


## **Figure S8. Structure prediction of psysol 3 using AfCycDesign tool.**

**A.** AfCycDesign tool, a ColabFold version adopted to predict structures of backbone cyclic peptides, was utilized to provide a prediction model for psysol 3. The predicted LDDT and predicted aligned error (PAE) metrics for the best model are provided. **B.** The metrics LDDT and PAE **(C)** for four models predicted are shown. It was recognized that the unique loop 6 sequence had the lowest prediction confidence. **D.** The predicted structure of psysol 3 was aligned with the published structure of kalata B1 (1NB1) and RMSD calculated with the commands ‘super’, RMSD = 1.09 Å (147 to 147 atoms), or ‘align’, RMSD = 1.83 Å (157 to 157 atoms), in pymol molecular graphic system v3.1.6.


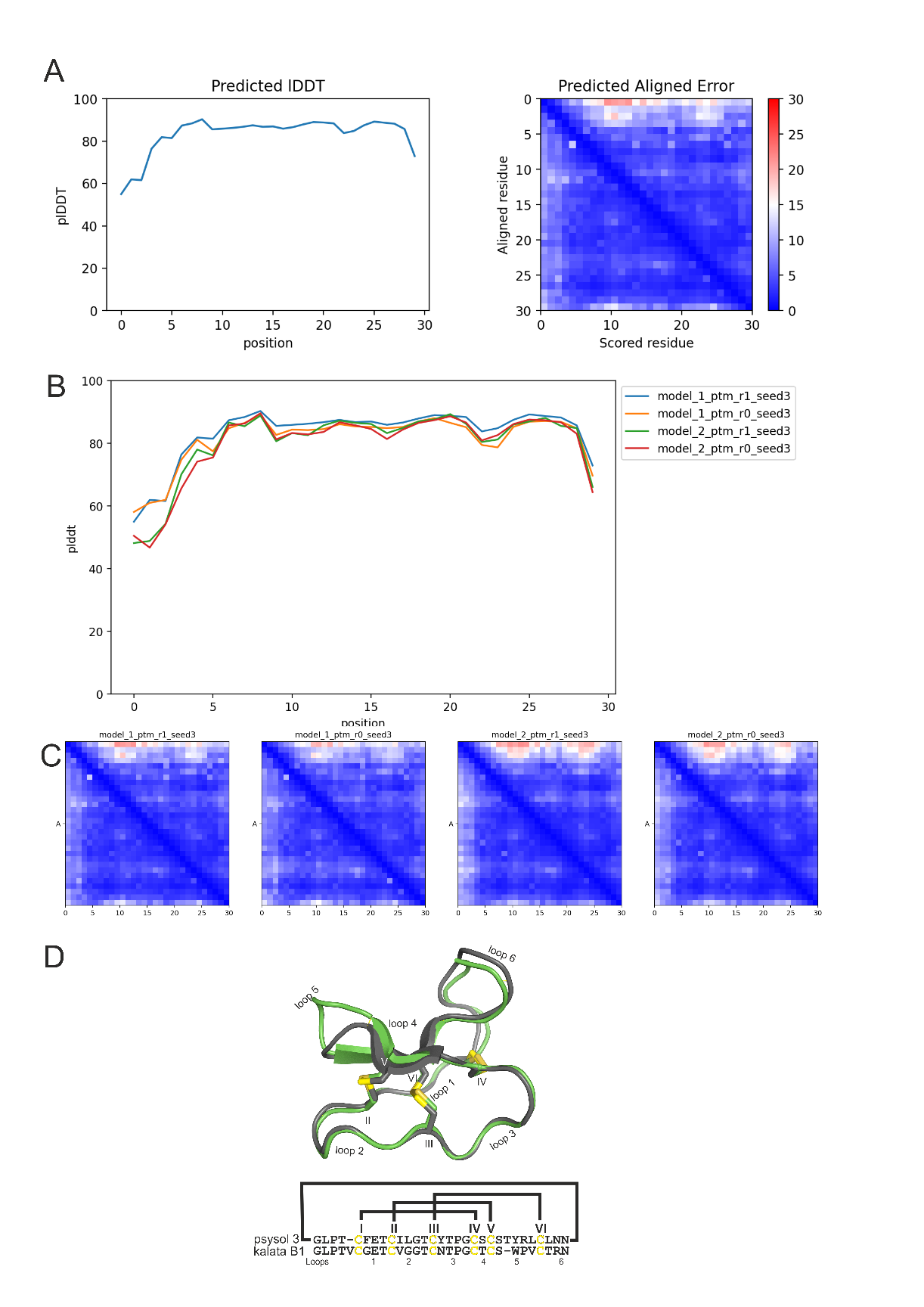

Supplement: Supplementary file 1 — Additional file 1. [file 13659_2026_598_MOESM1_ESM.docx]
